# NOP2-mediated 5-methylcytosine Regulates Lipid Metabolism Reprogramming to Prime Tumors for Ferroptosis in Bladder Cancer Progression

**DOI:** 10.7150/ijbs.126325

**Published:** 2026-04-16

**Authors:** Cheng Cheng, Jianguo Gao, Runzhe Wang, Yunfei Wu, Yupeng Wang, Baiye Jin, Guanghou Fu

**Affiliations:** 1 Department of Urology, The First Affiliated Hospital, School of Medicine, Zhejiang University, Hangzhou 310009, China.; 2 Zhejiang Engineering Research Center for Urinary Bladder Carcinoma Innovation Diagnosis and Treatment, Hangzhou 310024, China.; 3 Department of Urology, First Affiliated Hospital of Huzhou University, Huzhou, Zhejiang, China.; 4 Huzhou Key Laboratory of Precise Diagnosis and Treatment of Urinary Tumors, Huzhou, Zhejiang, China.

**Keywords:** NOP2, 5-methylcytosine, SCD, lipid metabolism reprograming, ferroptosis sensitivity.

## Abstract

RNA 5-methylcytosine (m^5^C) is an emerging epitranscriptomic modification implicated in the progression of multiple cancers, yet the landscape of m^5^C in bladder cancer (BCa) and underlying regulatory mechanisms remains largely elusive. In this study, we identified NOP2, an RNA methyltransferase, as a key driver of BCa progression. NOP2 was significantly upregulated in BCa tumors and associated with poor prognosis. Functionally, NOP2 promoted cell proliferation and invasion, enhancing tumor growth in xenograft models. Mechanistically, NOP2-mediated m^5^C deposition facilitates the recruitment of the m^5^C reader YBX1, thereby stabilizing *SCD* mRNA and boosting SCD expression. The NOP2/SCD axis orchestrated lipid metabolism reprogramming, altering the distribution of saturated, monounsaturated, and polyunsaturated fatty acids to suppress lipid peroxidation and protect BCa cells from ferroptotic stress. Collectively, our findings determine the oncogenic role of the m^5^C methyltransferase NOP2 in BCa and reveal a novel NOP2/YBX1/SCD axis that links epitranscriptomic regulation, metabolic reprogramming and ferroptosis evasion, providing a new target for therapeutic intervention.

## Introduction

Bladder cancer (BCa) is a major global health burden with high incidence, frequent recurrence and low survival rates in advanced patients 1-3. Although standard therapies for initial treatment involve surgery, chemotherapy, immunotherapy, and targeted agents, outcome for advanced diseases remains unfavorable, with a 5-year survival rate of 8% in metastatic cases 3. Revealing the drivers of tumor development and metastasis is essential for improving treatment of BCa.

Substantial evidence now establishes epitranscriptomic dysregulation and metabolism reprogramming as hallmarks of cancer 4-11. A key focus of recent research has been the critical role of RNA modification 5-methylcytosine (m^5^C) across a wide spectrum of cancers 5-8. As a pivotal "writer" enzyme depositing m^5^C modifications on RNA, the methyltransferase NOP2 has been implicated in tumor progression 12, 13. However, its specific role and mechanistic underpinnings in bladder cancer progression remain unclear.

Reprogrammed lipid metabolism of cancer cell not only supplies cells energy, but also plays an important role in ferroptosis 9, 10. Fatty acid desaturation serves multiple functions preventing lipo-toxicity, inhibiting ferroptosis, and regulating membrane permeability. The key enzyme Stearoyl-CoA desaturase (SCD) catalyzes the production of monounsaturated fatty acids from saturated precursors, playing a central role in maintaining lipid homeostasis. While SCD1 has been implicated in tumor progression and drug resistance across various cancers 14-16, the role of SCD dysregulation in bladder cancer is still not well defined.

In this study, we demonstrated that the m^5^C methyltransferase NOP2 is upregulated in bladder cancer and plays an oncogenic role through underlying molecular mechanism. We found that NOP2 likely drives metabolism reprogramming primarily by maintaining SCD expression in an m^5^C-dependent manner, leading to the shift of phospholipid composition and increased sensitivity to ferroptosis in BCa cells. Furthermore, we found that the suppression of NOP2/SCD axis significantly impaired tumor progression, and sensitize BCa cells to cisplatin as well. In summary, our results identify a novel NOP2/YBX1/SCD signaling axis linking epitranscriptomic remodeling, metabolic reprogramming and ferroptosis in BCa.

## Methods

### Clinical specimens

This study was conducted in accordance with medical ethics guidelines and was approved by the Institutional Ethics Committee of the First Affiliated Hospital, Zhejiang University School of Medicine (IIT20210265B-R1). A total of 103 pairs of bladder cancer (BCa) and matched adjacent normal tissues were procured from the same institution between January 2022 and January 2024. Informed consent was obtained from the patients. The clinicopathological characteristics of the cohort are summarized in Supplementary Table S1. Additionally, transcriptomic data and corresponding clinical information for the TCGA-BLCA cohort were retrieved from the TCGA data portal (https://portal.gdc.cancer.gov).

### Cell culture

The human embryonic kidney 293 T cells (HEK293T) cell line and two human BCa cell lines, T24 and UMUC3, were acquired from the Chinese Academy of Science (Shanghai, China). All cells were cultured in required medium supplied with 10% Fetal Bovine Serum (FBS; Gibco, cat. #A5256701) and 1% Penicillin/Streptomycin (Procell, cat. #PB180120) at 37℃ in a 5 % CO_2_ atmosphere.

### Animal studies

All animal procedures were conducted in an accordance with guidelines approved by the Institutional Ethics Committee of the First Affiliated Hospital, Zhejiang University School of Medicine. Mice were purchased from Zhejiang Animal Center and housed under specific-pathogen-free facility conditions. For xenograft tumor formation, 5 × 10^6^ T24 cells suspended in Matrigel (MCE; cat. #HY-K6001) were subcutaneously (s.c.) injected into 6-week-old male BALB/c nude mice. Ferrostatin-1 (Fer-1; MCE, cat. #HY-100579) was administered intraperitoneally (i.p.) at a dose of 5 mg/kg every 3 days for 2 weeks. The dose and dosing schedule used with CAY10566 (MCE, cat. #HY-15823), cisplatin (MCE, cat. #HY-17394) or vehicle controls was 5 mg/kg administered i.p. at 3-4day intervals. Tumor dimensions were measured using caliper, and tumor volume was determined based on the formula: V = (Length × Width × Height)/2.

### RNA interference, lentiviral infection and plasmid transfection

Small interfering RNAs (siRNAs; sequence listed in Supplementary Table S2) were synthesized by SUNYA Company (Hangzhou, China). Lentiviral vectors were generated by GeneChem (Shanghai, China). Short hairpin RNAs (shRNAs; target sequence listed in Supplementary Table S2) were cloned into the GV492 vector. Lentiviral infection was carried out following established protocols 13. Overexpression plasmids were obtained from Transheep Company (Shanghai, China). Mutations in the NOP2 gene were introduced using overlap extension PCR. Transfection of siRNAs or plasmids was performed using Lipofectamine 3000 (Invitrogen, cat. #L3000015) according to the manufacturer's instructions.

### RNA extraction and RT-qPCR

Total RNA was extracted from cultured cells and tissue samples using TRIzol reagent (Life Technologies, Austin, USA). cDNA was synthesized using the HiScript III RT SuperMix for qPCR (+gDNA wiper) kit (Vazyme, cat. #R323-01) according to the manufacturer's protocol. Quantitative polymerase chain reaction (qPCR) was then carried out with ChamQ Universal SYBR qPCR Master Mix (Vazyme, cat. #Q711-02). Gene expression levels were quantified via the 2^^-ΔΔ^CT method, with GAPDH mRNA serving as the internal normalization control. The sequences of all primers used are provided in Supplementary Table S3.

### Western blot

Western blot analysis was performed according to previously described methods 13. Primary and secondary antibodies used are listed in Supplementary Table S6. Protein bands were visualized using an enhanced chemiluminescence detection system (Beyotime, cat. #P0018S).

### Dot blot

Total RNA was extracted from treated cells and resuspended in three volumes of RNA incubation buffer, followed by denaturation at 65 °C for 5 minutes. Different amounts of RNA (400, 200, and 100 ng) were applied to Amersham Hybond N^+^ membranes (GE Healthcare, USA) using a Bio-Dot apparatus (Bio-Rad, USA) with a mixture of 20 × SSC buffer (pre-cooled at 4 ℃; Sigma-Aldrich, cat. #SRE0068). The membrane was UV cross-linked at 254 nm for 5 minutes on the both sides. To verify equal RNA loading, the membrane was stained with 0.02% methylene blue in 0.3 mol/L sodium acetate and scanned. After blocking with 5% BSA, the membrane was incubated with an anti-5-methylcytosine (5-mC) antibody, followed by an HRP-conjugated anti-rabbit secondary antibody. Signal detection was performed using an imaging system.

### Immunohistochemistry

Immunohistochemical (IHC) staining was performed based on established protocols 13. Briefly, tissue sections were incubated with specific primary antibodies, followed by HRP-conjugated secondary antibodies. The sections were counterstained with hematoxylin and imaged under an inverted microscope. All antibodies used are listed in Supplementary Table S4.

The IHC score was calculated by multiplying the staining intensity score by the proportion of positive cells. Staining intensity was graded as follows: 0 (negative), 1 (weak), 2 (moderate), and 3 (strong). The percentage of positive cells was scored as: 0 (0%), 1 (< 10%), 2 (10%-50%), 3 (50%-80%), and 4 (> 80%). All scoring was performed independently by two experienced pathologists.

### Cell proliferation assays

#### CCK-8 assay

Cells were seeded at a density of 2 × 10^3^ per well in 96-well plates. At designed time points, CCK-8 reagent (MCE, cat. #HY-K0301) was added to each well. After 1 hour of incubation, the absorbance at 450 nm were detected.

#### EdU assay

A total of 2 × 10^4^ cell per well were planted in 96-well plates and incubated with 50 µM EdU (UElandy, cat. #C6046M) for 2 hours. Subsequently, cells were fixed with 4% paraformaldehyde, permeabilized with 0.1% Triton X-100 for 10 minutes, and stained with EdU YF® 488 Azide for 30 minutes. Nuclei were stained with Hoechst 33342 for 30 minutes.

#### Colony formation assay

Cells were plated in 6-well plates at 2 × 10³ cells per well and cultured for 7 days. Following incubation, cells were fixed with 4% paraformaldehyde and stained with 0.1% crystal violet.

### Transwell and wound healing assays

#### Transwell assay

A total of 3 × 10⁴ cells suspended in serum-free medium were seeded into the upper chamber of an 8μm Transwell insert (Corning, Washington DC, USA) and cultured for 24 hours. Cells that invaded through the membrane to the lower surface were fixed with 4% paraformaldehyde, stained with 0.1% crystal violet and counted under microscope.

#### Wound healing assay

Cells were grown in 6-well plates until they reached 90% confluence. A uniform wound was created in each well using a 200 μL sterile pipette tip. The same wound areas were imaged at specified time points to monitor cell migration.

### RNA sequencing (RNA-Seq)

Total RNA was extracted from *NOP2*-knockdown and control T24 cells. Sequencing libraries were prepared and RNA sequencing was performed by OE Biotech (Shanghai, China) on the Illumina HiSeq X Ten platform with PE150 configuration. Differentially expressed genes (DEGs) were identified using a threshold of | log₂ (fold change) | > 1.5 and a q-value < 0.05. Functional annotation of DEGs was carried out through KEGG pathway enrichment analysis.

### RNA bisulfite sequencing (RNA-Bis-Seq)

RNA-Bis-Seq of m^5^C modification detection was performed by CloudSeq Biotech Inc. (Shanghai, China). Total RNA was extracted from *NOP2*-knockdown and control T24 cells. Following ribosomal RNA (rRNA) depletion, the RNA was subjected to bisulfite conversion and purification. Sequencing libraries were constructed and sequenced on an Illumina HiSeq 4000 system using 150 bp paired-end reads. After trimming 3′ adaptors and filtering low-quality reads, clean reads were aligned to the UCSC HG19 reference genome using meRanGh. Methylated sites were called with meRanCall, and differential methylation was identified using meRanCompare. The m^5^C sites were annotated based on Ensembl genome features, and their distribution was visualized with the MetaPlot package in R.

### RNA stability assay

BCa cells were transfected with the indicated vectors and then treated with 5 µg/mL actinomycin D (MCE, cat. #HY-17559) for specified duration. After treatment, total RNA was extracted and analyzed by RT-qPCR. The half-life of the target mRNA was calculated using non-linear regression analysis.

### RNA immunoprecipitation (RIP)

The RIP assay was performed using the Magna RIP™ RNA-Binding Protein Immunoprecipitation Kit (Millipore, Burlington, USA) according to the manufacturer's instructions. Briefly, approximately 1 × 10⁷ cells were collected and lysed in RIP lysis buffer. Magnetic beads conjugated with 5 μg of antibody against the target protein or normal IgG were incubated with the cell lysates overnight at 4 °C. The immunoprecipitated RNA-protein complexes were then treated with proteinase K buffer to digest proteins. RNA was extracted using phenol-chloroform purification and analyzed by RT-qPCR. Results were normalized to the input sample.

### RNA pulldown

The RNA pulldown assay was performed with the Pierce Magnetic RNA-Protein Pull-Down Kit (Thermo Fisher Scientific, cat #20164). 3'-labeled biotin-labeled RNA probes, including 50-bp *SCD* RNA sequences with m^5^C modification (SCD [m^5^C]) or without m^5^C modification (SCD [C]) at the same site, were synthesized (as detailed in Supplementary Table S4) and then incubated with total protein extracts from BCa cells. In brief, 50 pmol biotinylated RNA probes were incubated with 50 μL of streptavidin beads for 30 min. Then, the mix was incubated with 2 mg of protein lysates at 4℃ for 60min. Following the pulldown assay, nonspecific signals were removed by washing throughout. Protein samples were eluted by boiling with SDS buffer and subjected to western blot using anti-YBX1 antibodies.

### Luciferase reporter gene assays

The wild-type (*SCD*^WT^) or mutant (*SCD*^MUT^) 3'-UTR sequence of SCD, containing a cytosine-to-guanine substitution at the predicted m^5^C site, was cloned into a reporter vector constructed by Tran sheep Company (Shanghai, China). The resulting constructs were co-transfected with the pRL-SV40 Renilla luciferase control vector into BCa cells seeded in 24-well plates. After 48 hours, luciferase activity was measured using the Dual-Luciferase Reporter Assay Kit (Vazyme, Nanjing, China). Firefly luciferase activity was normalized to that of Renilla luciferase for each sample.

### Lipid quantification assay

BCa cells with the indicated treatments were collected and washed with PBS. Lipids were extracted from cells using a Lipid Extraction Kit (Cell Biolabs, cat. #STA-612). The intracellular unsaturated fatty acids (UFAs) content was detected at OD 540 nm, and the values were calculated according to the Lipid Quantification Kit (unsaturated fatty acids; Cell Biolabs, cat. #STA-613) protocol.

### Subcellular fractionation

Subcellular fractionation was carried out with the PARIS™ kit (Invitrogen, cat #AM1921) according to the manufacturer's protocol. In brief, about 5 × 10⁶ collected cells were washed in cold PBS and lysed on ice for 10 min using 400 µl of ice-cold cell fractionation buffer. Following 500 × g centrifugation for 5 min, the supernatant (cytoplasmic fraction) was transferred to an RNase-free tube. The pellet was then lysed in an equal volume of cell disruption buffer and homogenized by vortex for 40 min on ice. After 12,000 × g centrifugation for 5 min, the supernatant (nuclear fraction) was collected. Cytoplasmic and nuclear RNAs were then extracted for RT-qPCR analysis.

### Untargeted lipidomics assay

Approximately 1.2 × 10^7^ cells were collected and washed twice with 1 ml of 0.9% normal saline. Then, 800 μl of pre-cooled methanol (-80 °C) and 320 μl ice-cold water were added, and the mixture was homogenized by shaking for 10 seconds. Metabolites were scraped and transferred to a 1.5 mL centrifuge tube. After adding 800 μL of pre-cooled chloroform, the sample was centrifuged at 14,000 × g and 4 °C for 15 minutes. A 700 μL aliquot of the lower organic phase was transferred to a new tube and dried under nitrogen gas at room temperature. The dried lipid extracts were reconstituted in 120 μL of solvent (chloroform:methanol:water, 6:3:0.5, v/v/v), vigorously vortexed for 5 minutes, and centrifuged at 14,000 × g for 5 minutes at room temperature. Finally, 100 μL of the supernatant was transferred to a 2 mL mass spectrometry vial containing internal standards for LC-MS/MS analysis. Liquid chromatography tandem mass spectrometry (LC-MS/MS) and subsequent data processing were performed by OE Biotech (Shanghai, China).

### Flow cytometry

Lipid peroxidation was assessed using 1 μmol/L C11-BODIPY 581/591 (Beyotime, cat. #S0043M), and cell viability was evaluated using propidium iodide (PI, Invitrogen, cat. #P1304MP), both according to the manufacturers' protocols. Data acquisition was performed on a CytoFLEX flow cytometer (Beckman Coulter), and results were analyzed with FlowJo software, with a minimum of 1 × 10⁴ cells acquired per condition.

### Transmission electron microscopy

Transmission electron microscopy was performed by the High-Resolution Electron Microscopy Facility at the Instrument Center of Biossci Biotechnology Co., Ltd (Wuhan, China). T24 and UMUC3 cells were cultured under indicated conditions, trypsinized, and fixed in 2.5% glutaraldehyde. The cells were then post-fixed in 1% osmium tetroxide containing 0.1% potassium ferricyanide, dehydrated through a graded ethanol series (50% to 100%), and embedded in epoxy resin. Ultrathin sections (60-80 nm) were prepared using a ultramicrotome. Sections were stained with 2% uranyl acetate in saturated alcoholic solution and lead citrate, and imaged under an 80 kV transmission electron microscope (HITACHI, Tokyo, Japan).

### Cellular Fe^2+^ content assay

Intracellular Fe²⁺ levels were measured using the fluorescent indicator FerroOrange (DojinDo, cat. #F374) following the manufacturer's protocol. Briefly, *NOP2*-knockdown BCa cells were seeded at a density of 5 × 10³ cells per well in 96-well plates. After incubation with 1 μM FerroOrange for 30 minutes at 37 °C, the cells were washed 3 times with PBS. Fluorescence was then measured using a Synergy Neo2 microplate reader (BioTek, Winooski, USA) at an excitation/emission wavelength of 543 nm.

### Intracellular ROS level assay

Intracellular ROS levels were measured using a ROS Assay Kit (Beyotime, cat. #S0033S) according to the manufacturer's instructions. Briefly, 5 × 10³ cells were seeded per well in a 96-well plate and incubated with DCFH-DA probe for 1 hour at 37 °C. Fluorescence intensity was measured at excitation/emission wavelengths of 490 nm and 525 nm, respectively, using a Synergy Neo2 microplate reader (BioTek, Winooski, USA).

### Survival analysis

RNA-seq data and corresponding clinical information for GSE and TCGA-BLCA cohorts was downloaded and processed. Overall Survival (OS), Progression-Free Survival (PFS), Disease-Free Survival (DFS), and Disease-Specific Survival (DSS) were evaluated, with a maximum follow-up time set at five years (60 months). Patients were stratified into high- and low-expression groups for each gene based on an optimal cutoff value calculated through “survminer” package. The Kaplan-Meier method was used to generate survival curves for all four endpoints, and the log-rank test was applied to compare survival differences between the two groups.

### Statistical analyses

All statistical analyses were conducted using GraphPad Prism 10.0, SPSS 20.0, and R version 4.3.3 (https://www.r-project.org/). Quantitative data were expressed as mean ± standard deviation (SD). Comparisons between groups for continuous variables were performed using one-way ANOVA or two-tailed Student's t-test, as appropriate. Categorical variables were compared using the Mann-Whitney U test. Correlations were evaluated by Pearson's correlation analysis. A P-value less than 0.05 was considered statistically significant, with significance levels indicated as follows: *P < 0.05, **P < 0.01, and ***P < 0.001.

## Results

### NOP2 is upregulated in BCa tissues and drives tumor progression

**Figure 1:**
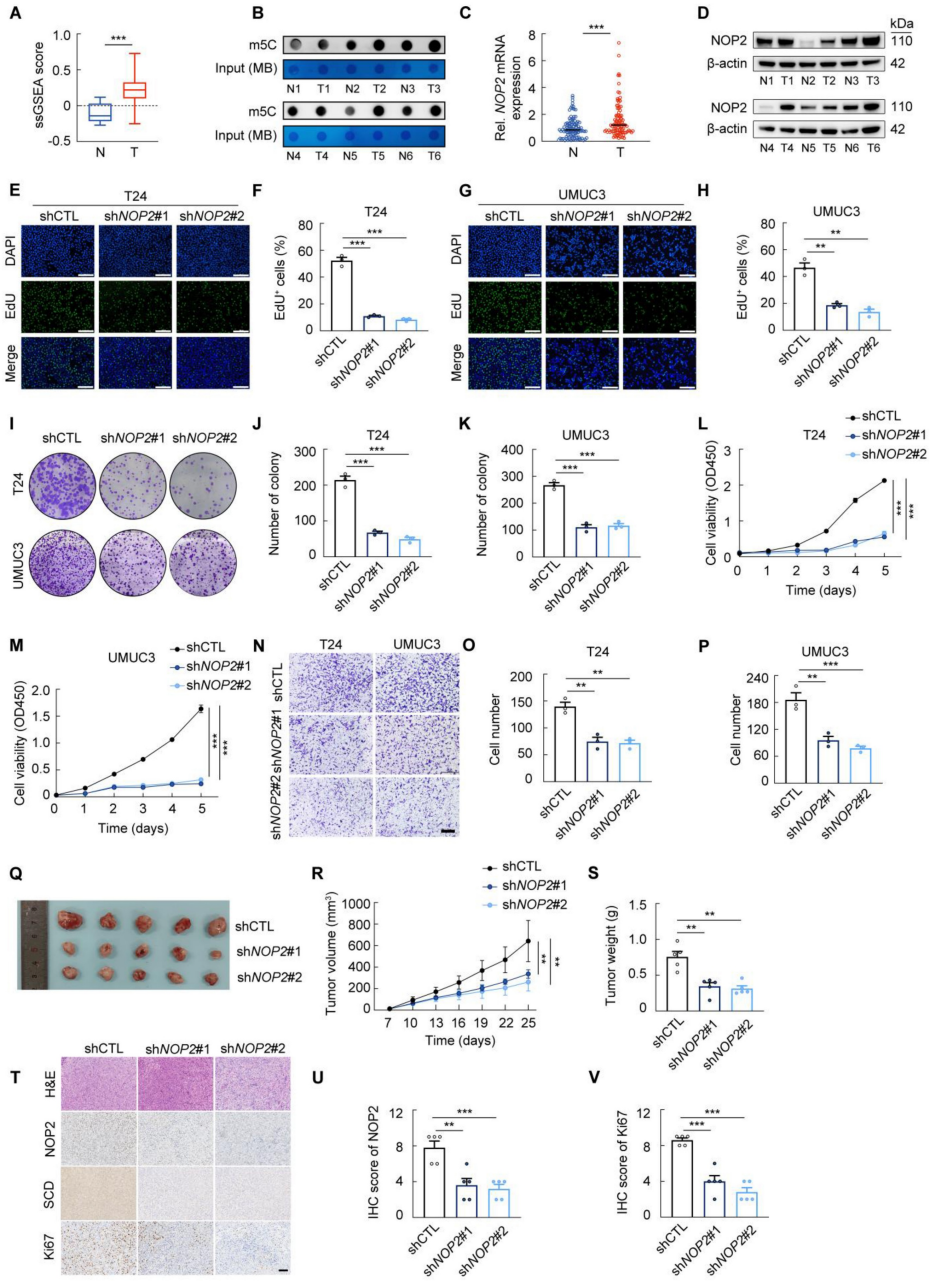
** NOP2 is upregulated in BCa tissues and drives tumor progression (A)** m^5^C scores between BCa tissues and paracancerous tissues from the TCGA database. **(B)** Dot blot analysis of RNA m^5^C levels in tumor and paracancerous tissues from BCa patients. **(C)** RT-qPCR analysis of* NOP2* mRNA levels in tumor and paracancerous tissues from BCa patients. **(D)** Western blot analysis of NOP2 protein levels in tumor and paracancerous tissues from BCa patients. **(E-H)** Edu assay in *NOP2*-knockdown T24 and UMUC3 cells **(E, G)**, and the quantification of Edu^+^ cells were shown in **(F, H)**. **(I-K)** Colony formation assay of *NOP2*-knockdown T24 and UMUC3 cells **(I)**, and the quantification of clones were shown in **(J, K)**. **(L, M)** CCK-8 assay in *NOP2*-knockdown T24 and UMUC3 cells. **(N-P)** Transwell assay of *NOP2*-knockdown T24 and UMUC3 cells (N), and the quantification of trans-membrane cells were shown in **(O, P)**. **(Q-S)** Xenograft tumors of T24 or *NOP2*-knockdown T24 cells in BALB/c nude mice **(Q)**. Growth kinetics of the formed tumors were monitored and examined **(R)**. Tumor weights of the isolated tumor were analyzed **(S)**. **(T-V)** Representative immunohistochemical staining of NOP2, Ki67 and SCD in xenograft tumors **(T)**, with corresponding immunoreactive scores quantified for NOP2 **(U)** and Ki67 **(V)**. Statistical analysis was performed using a one-way analysis of variance (ANOVA), followed by Dunnett's test **(F-K, O-P, S-V)**, repeated measures ANOVA followed by Mauchly's test of sphericity **(L-M, R)**, or unpaired two-tailed student t-test **(A, C)**. Data are presented as means ± standard deviation; *p < 0.05, **p < 0.01, ***p < 0.001, n.s. non-significant.

Growing evidence indicates that m^5^C modification plays a critical role in tumor progression 17-19. To quantify RNA methylation levels in BCa, we employed an m^5^C score derived from 18 m^5^C regulators 20. Our analysis revealed a significant upregulation of m^5^C scores in TCGA-BLCA tumor tissues compared to their paracancerous counterparts (Fig. 1A). This finding was validated in local BCa samples, which exhibited significantly elevated RNA m^5^C levels (Fig. 1B). Analysis of multiple GSE profiles and TCGA-BLCA data further revealed distinct expression patterns of key m^5^C methyltransferases between tumor and adjacent normal tissues (Supplementary Fig. S1A). Notably, NOP2 displayed a marked differential expression in BCa tumors versus paired tissues, leading us to prioritize it for further study. Consistently, RT-qPCR and Western Blot analysis of matched patient specimens confirmed that NOP2 is significantly upregulated in BCa tumors compared to adjacent non-cancerous tissues (Fig. 1C-D). Moreover, high NOP2 expression was strongly associated with poor prognosis (Supplementary Fig. S1B-C). Clinicopathological stratification further revealed significant correlations between NOP2 expression level and tumor size, pathological grade, and T stage (Supplementary Fig. S1D). Collectively, these results identify NOP2 as a differentially expressed and prognostically significant m^5^C regulator in BCa.

To further investigate the oncogenic role of NOP2 in BCa, we established two *NOP2*-knockdown cell lines using distinct lentiviral shRNAs (Supplementary Fig. S1E). Subsequent cell proliferation and invasion assays revealed that *NOP2* knockdown significantly suppressed the malignant phenotype of T24 and UMUC3 cells *in vitro* (Fig. 1E-P; Supplementary Fig. S1F-I). To validate these findings *in vivo*, we established a subcutaneous xenograft model. Tumors derived from *NOP2*-knockdown T24 cells showed significantly reduced growth and weight compared to controls (Fig. 1Q-S). Consistently, IHC analysis confirmed a lower proliferation index, as indicated by weaker Ki67 staining in the NOP2-knockdown group (Fig. 1T-V).

Collectively, our findings demonstrate that NOP2 is upregulated in BCa and acts as a pro-oncogenic factor driving tumor progression, although its precise molecular mechanisms warrant further elucidation.

### NOP2 promotes SCD expression via an m^5^C-dependent manner in bladder cancer

**Figure 2:**
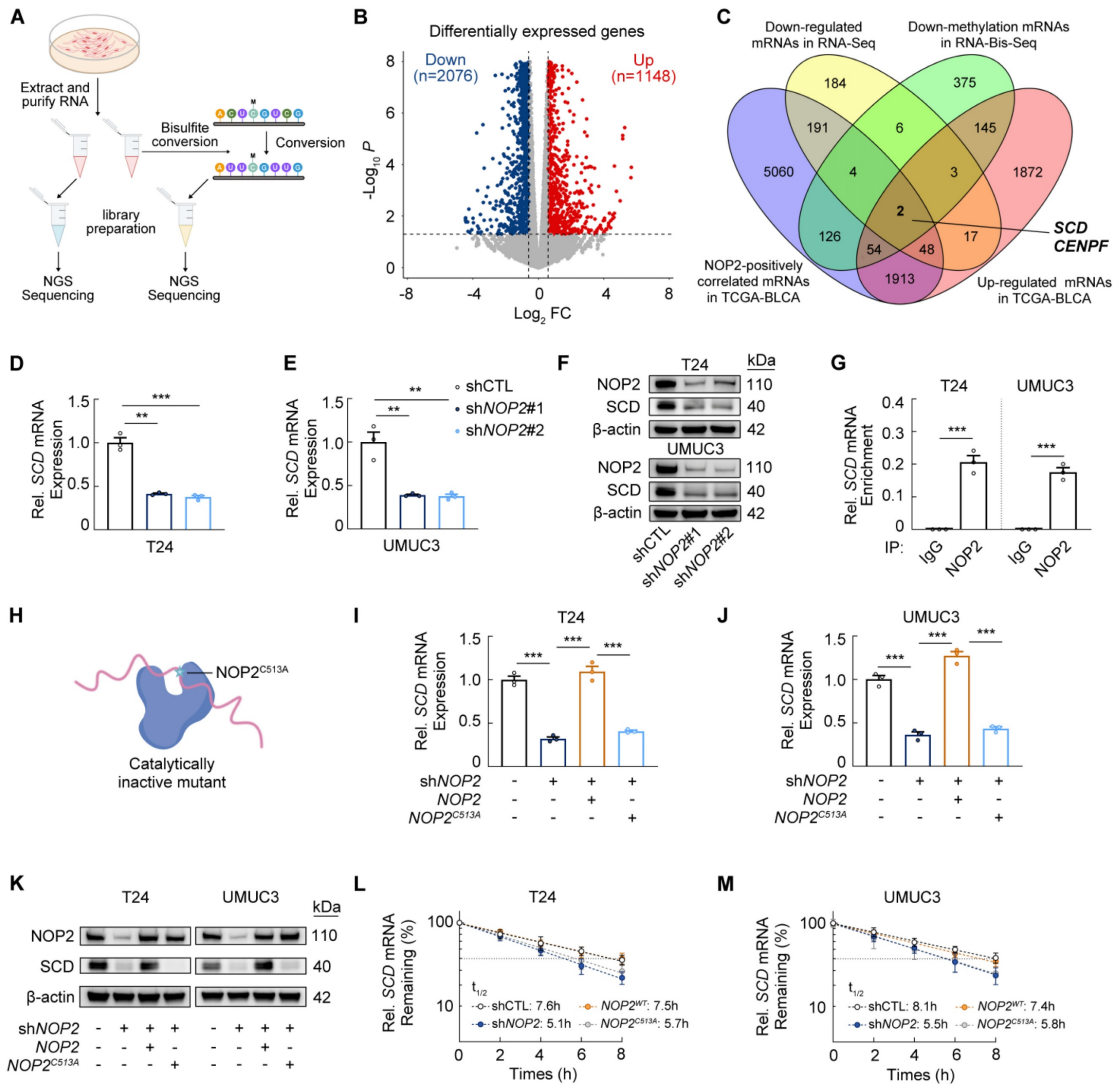
** NOP2 promotes SCD expression via an m^5^C-dependent manner in bladder cancer (A)** Schematic representation of the process of RNA-Seq and RNA-Bis-Seq in BCa cells. **(B)** Heatmap displaying the differentially expressed genes between the *NOP2*-knockdown BCa cells and the controls. **(C)** Transcripts that were positively correlated with NOP2, upregulated in TCGA-BLCA tumors versus adjacent tissues, and exhibited reduction in both m^5^C methylation and expression levels upon *NOP2* knockdown in T24 cells **(D, E)** *SCD* mRNA levels in *NOP2*-knockdown BCa cells and the controls. **(F)** Western blot analysis of SCD protein levels in BCa cells following *NOP2* knockdown and the controls. **(G)** RIP-qPCR analysis of binding ability of NOP2 to *SCD* mRNA in T24 and UMUC3 cells. **(H)** Cartoon depicting that NOP2C513A mutation blocks the methyltransferase activities of NOP2. **(I, J)** *SCD* mRNA levels in BCa cells following the treatment with indicated vectors. **(K)** Western blot analysis of SCD protein levels in BCa cells following the treatment with indicated vectors. **(L, M)** Analysis of SCD mRNA half-life in T24 and UMUC3 cell lines following the treatment with indicated vectors. Statistical analysis was performed using a one-way ANOVA, followed by Dunnett's test **(D-E, I-J)**, or unpaired two-tailed student t-test **(G)**. Data are presented as means ± standard deviation; *p < 0.05, **p < 0.01, ***p < 0.001, n.s. non-significant.

Since NOP2 functions as an m^5^C methyltransferase mediating methylation of diverse RNA species, we investigated its m^5^C-dependent role in BCa progression. RNA-Seq and RNA-Bis-Seq were performed on *NOP2*-knockdown cells and control cells (Fig. 2A). RNA-Seq analysis identified 1148 up-regulated and 2076 down-regulated genes (Fig. 2B), while RNA-Bis-Seq analysis revealed that *NOP2* knockdown significantly altered global m^5^C methylation across multiple mRNA regions (Supplementary Fig. S2A-C). Integrated analysis of RNA-Seq, RNA-Bis-Seq, and BLCA-TGCA data demonstrated that *NOP2* knockdown in BCa cells reduced both m^5^C methylation and mRNA expression of *SCD* and *CENPF* relative to controls (Fig. 2C). RT-qPCR validation in the other cell line confirmed that *NOP2* knockdown significantly reduced *SCD* mRNA, but not *CENPF* mRNA (Fig. 2D-E; Supplementary Fig. S2D). Consistently, *NOP2* knockdown reduced SCD protein levels (Fig. 2F; Supplementary Fig. S2D). Notably, SCD was significantly elevated in BCa tissues (Supplementary Fig. S2F-G), and high *SCD* expression correlated with poor survival of BCa patients (Supplementary Fig. S2H-J).

Subsequently, RIP assays demonstrated that NOP2 antibody enriched *SCD* mRNA *in vitro* (Fig. 2G; Supplementary Fig. S2K). To determine whether the regulation of NOP2 upon *SCD* depends on its m^5^C methyltransferase activity, we generated a catalytically inactive NOP2 mutant (NOP2^C513A^) by disrupting the catalytic site to abrogate m^5^C methylation 21 (Fig. 2H; Supplementary Fig. S2L-M). Reintroduction of wild-type NOP2 (NOP2^WT^), but not NOP2^C513A^, restored *SCD* mRNA and protein levels in *NOP2*-knockdown BCa cells (Fig. 2I-K). Mechanistically, NOP2-mediated m^5^C modification likely promote SCD expression by enhancing mRNA stability. Consistent with this, actinomycin D assays showed a significantly shortened half-life of *SCD* mRNA in *NOP2*-knockdown BCa cells, which was rescued by NOP2^WT^, but not NOP2^C513A^ (Fig. 2L-M).

### YBX1 recognizes and stabilizes m^5^C modified *SCD* mRNA

**Figure 3:**
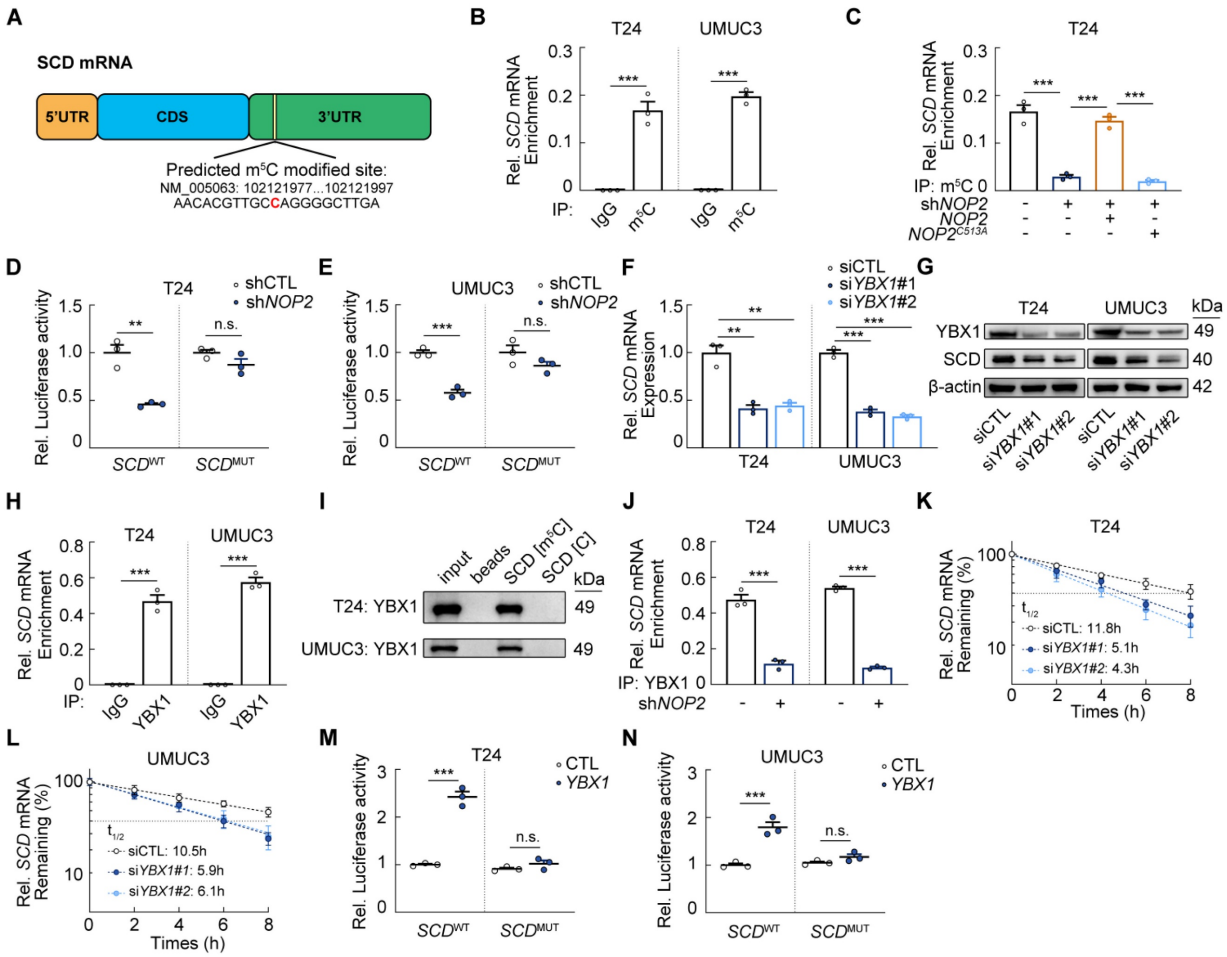
** YBX1 recognizes and stabilizes m^5^C modified *SCD* mRNA (A)** Diagram of the predicted m^5^C site in *SCD* mRNA. **(B)** RIP-qPCR analysis of m^5^C modification of *SCD* mRNA in T24 and UMUC3 cells. **(C)** RIP-qPCR analysis of m^5^C modification upon *SCD* mRNA in T24 cells following the treatment with indicated vectors. **(D, E)** Luciferase reporter assays of luciferase reporter gene containing the wild-type SCD-m^5^C site (SCD^WT^) or the mutant m^5^C site (SCD^MUT^) in *NOP2* knockdown T24 and UMUC3 cells. **(F)** *SCD* mRNA levels in *YBX1*-knockdown BCa cells and the controls. **(G)** Western blot analysis of SCD protein levels in *YBX1*-knockdown BCa cells. **(H)** RIP-qPCR analysis of binding ability of YBX1 to *SCD* mRNA in T24 and UMUC3 cells. **(I)** Western blotting analysis of potential m^5^C modified *SCD* mRNA motif binding protein YBX1 obtained from RNA pulldown. **(J)** RIP-qPCR analysis of binding ability of YBX1 to SCD mRNA in *NOP2*-knockdown BCa cells and the controls. **(K, L)** Analysis of *SCD* mRNA half-life in *YBX1*-knockdown T24 and UMUC3 cell lines. **(M, N)** Luciferase reporter assays of luciferase reporter gene containing the wild-type SCD-m^5^C site (SCD^WT^) or the mutant m^5^C site (SCD^MUT^) in YBX1 knockdown T24 and UMUC3 cells. Statistical analysis was performed using a one-way ANOVA, followed by Dunnett's test **(C, F)**, or unpaired two-tailed student t-test **(B, D-E, H-N)**. Data are presented as means ± standard deviation; *p < 0.05, **p < 0.01, ***p < 0.001, n.s. non-significant.

We then hypothesized that NOP2 directly catalyzes m^5^C deposition on *SCD* mRNA. RNA-Bis-Seq analysis identified an unconverted m^5^C site in the 3'-UTR of *SCD* mRNA (Fig. 3A). We next performed RNA immunoprecipitation (RIP) assays, demonstrating that the m^5^C antibody could enrich *SCD* mRNA *in vitro* (Fig. 3B; Supplementary Fig. S3A), whereas the enrichment of *SCD* mRNA was significantly reduced after silencing NOP2 (Fig. 3C; Supplementary Fig. S3B). More specifically, this effect was prominently reversed by ectopic expression of wild-type NOP2, while inactive NOP2 mutant failed to produce the same effect (Fig. 3C). In addition, we then measure the activity of a luciferase reporter fused to *SCD*-3'-UTR (*SCD*^WT^) or *SCD*-MUT-3'-UTR (*SCD*^MUT^). The luciferase activity of *SCD*^WT^ was markedly increased in *NOP2* knockdown cells, with no such effect observed for *SCD*^MUT^ (Fig. 3D-E).

A key function of m^5^C modification is to recruit dedicated “reader” proteins such as YBX1 and ALYREF, which recognize the modification to promote mRNA stability or nuclear export 22-25. We investigated whether YBX1 or ALYREF mediates *SCD* mRNA regulation in an m^5^C-dependent manner. YBX1 knockdown significantly reduced both *SCD* mRNA and protein levels (Fig. 3F-G; Supplementary Fig. S3C-D), whereas ALYREF deletion had no significant effect on *SCD* mRNA (Supplementary Fig. S3E-G). RIP-qPCR analysis confirmed specific YBX1 binding of m^5^C-modified *SCD* mRNA across different cell lines (Fig. 3H; Supplementary Fig. S3H), and RNA pulldown using m^5^C modified *SCD* probe further validated this interaction (Fig. 3I). Critically, NOP2 depletion abolished YBX1 binding to *SCD* mRNA (Fig. 3J; Supplementary Fig. S3I). Given the well-established role of YBX1 in stabilizing m^5^C-modified transcripts, actinomycin D assays showed *SCD* mRNA half-life was significantly shortened upon *YBX1* knockdown (Fig. 3K, L). Moreover, YBX1 overexpression significantly enhanced the luciferase activity of *SCD*^WT^ but not *SCD*^MUT^ (Fig. 3M-N). In addition, *YBX1* knockdown did not significantly alter nucleocytoplasmic shuttling of *SCD* mRNA (Supplementary Fig. S3J). Collectively, these results demonstrated that NOP2-mediated *SCD* mRNA stabilization occurs via YBX1-dependent mechanisms.

### Knockdown of *NOP2* decreases monounsaturated fatty acids dependent on SCD

**Figure 4:**
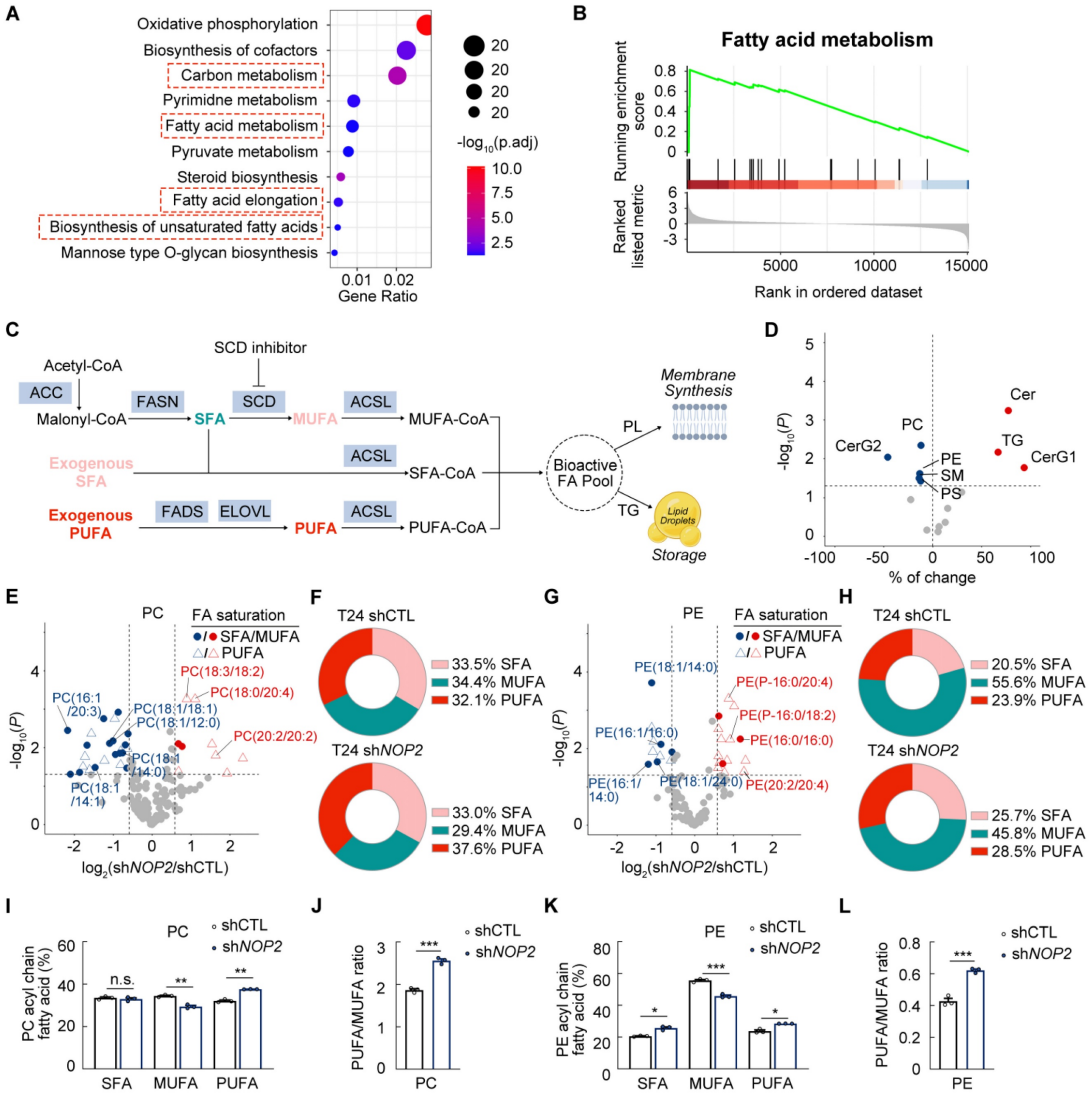
** Knockdown of *NOP2* decreases monounsaturated fatty acids dependent on SCD (A)** KEGG analysis of the DEG from the transcripts in *NOP2* knockdown T24 cells and the controls. **(B)** Enrichment analysis of the fatty acid metabolism signature in DEG analysis between *NOP2* knockdown T24 cells and the controls. **(C)** Schematic representation of FA synthesis pathways. **(D)** Global lipidomic alteration between NOP2 knockdown T24 cells and the controls. **(E-L)** Lipidomics showing the distribution of SFAs, MUFAs, and PUFAs in phosphatidylcholine **(PC; E, F, I)** and phosphoethanolamine **(PE; G, H, K)** in *NOP2* knockdown T24 cells compared with control. PUFA:MUFA ratio for PC and PE are shown in **(J, L)**. Statistical analysis was performed using an unpaired two-tailed student t-test **(I-L)**. Data are presented as means ± standard deviation; *p < 0.05, **p < 0.01, ***p < 0.001, n.s. non-significant.

To elucidate the underlying mechanism, we performed Kyoto Encyclopedia of Genes and Genomes (KEGG) and Gene Set Enrichment Analysis (GSEA), revealing that NOP2 depletion disrupts lipid metabolism in BCa cells (Fig. 4A-B). Fatty acid (FA) synthesis primarily produces saturated fatty acids (SFAs) and monounsaturated fatty acids (MUFAs), whereas polyunsaturated fatty acids (PUFAs) are largely obtained from dietary sources and undergo sequential enzymatic modifications: desaturation by FADS and elongation by ELOVL (Fig. 4C) 26, 27. Following ACSL-mediated activation, FAs enter the metabolically active pool and undergo biotransformation to become phospholipids (PLs) incorporated into plasma membranes, stored in lipid droplets, or oxidized in mitochondria (Fig. 4C) 28, 29. Given SCD catalyzes stearoyl-CoA to oleoyl-CoA conversion (Supplementary Fig. S4A), NOP2 depletion likely alters FA composition and lipid function via SCD suppression. Consistently, NOP2 knockdown decreased unsaturated FA contents in T24 and UMUC3 cells (Supplementary Fig. S4B).

To further determine the mechanism underlying *NOP2* knockdown-mediated lipid metabolic alterations, global lipidomic analysis was performed in *NOP2*-knockdown T24 cells and controls. Lipidomic profiling identified 704 distinct lipid species, including 131 phosphatidylcholines (PCs), 92 phosphatidylethanolamines (PEs), 168 triglycerides (TGs), and other lipid classes (Supplementary Fig. S4C). NOP2 depletion resulted in a global reduction of glycerophospholipid (GP) categories, specifically PCs, PEs, and phosphatidylserines (PSs) (Fig. 4D). Conversely, total TG content increased significantly in *NOP2*-knockdown BCa cells (Fig. 4D). Notably, the proportion of TGs containing monounsaturated fatty acid chains was markedly reduced (Supplementary Fig. S4D). Given lipid droplets serve as intracellular TG reservoirs, Oil Red O staining revealed increased lipid droplet numbers in *NOP2*-knockdown BCa cells (Supplementary Fig. S4E). In addition, *NOP2* knockdown elevated ceramides (CERs) while decreasing sphingomyelins (SMs) in BCa cell lines (Fig. 4D).

The fatty acyl chains of membrane phospholipids exhibit significant diversity in chain length and saturation, which critically govern cellular membrane biophysics and modulate membrane-dependent biological processes 30. PUFA-enriched PLs are highly susceptible to peroxidation by reactive oxygen species (ROS), triggering lipid peroxidation and ferroptosis 31. Conversely, MUFAs suppress this process by displacing PUFAs from plasma membrane PLs 32. We examined the role of NOP2 in phospholipid composition, especially focusing on SFA:MUFA:PUFA ratios. Lipidomic profiling revealed reduced MUFA incorporation into PLs, particularly PCs and PEs, alongside elevated PUFA levels after NOP2 depletion (Fig. 4E-K; Supplementary Fig. S4F-L). Consequently, *NOP2* knockdown increased the phospholipid PUFA:MUFA ratio in T24 cells (Fig. 4J, L; Supplementary Fig. S4M). These data indicate that NOP2 depletion impairs the conversion from SFA to MUFA. Notably, the 16:1(n-7)/16:0 and 18:1(n-9)/18:0 ratios, established proxies for SCD activity, were significantly diminished in NOP2-depleted cells versus controls (Supplementary Fig. S4N), confirming a strong correlation between NOP2 expression and SCD enzymatic activity. Collectively, these results demonstrate that NOP2 regulates lipid homeostasis in BCa cells by promoting MUFA generation and modulating PUFA storage/metabolism.

### Inhibition of SCD-dependent monounsaturated fatty acids biosynthesis sensitives BCa cells to ferroptotic cell death

**Figure 5:**
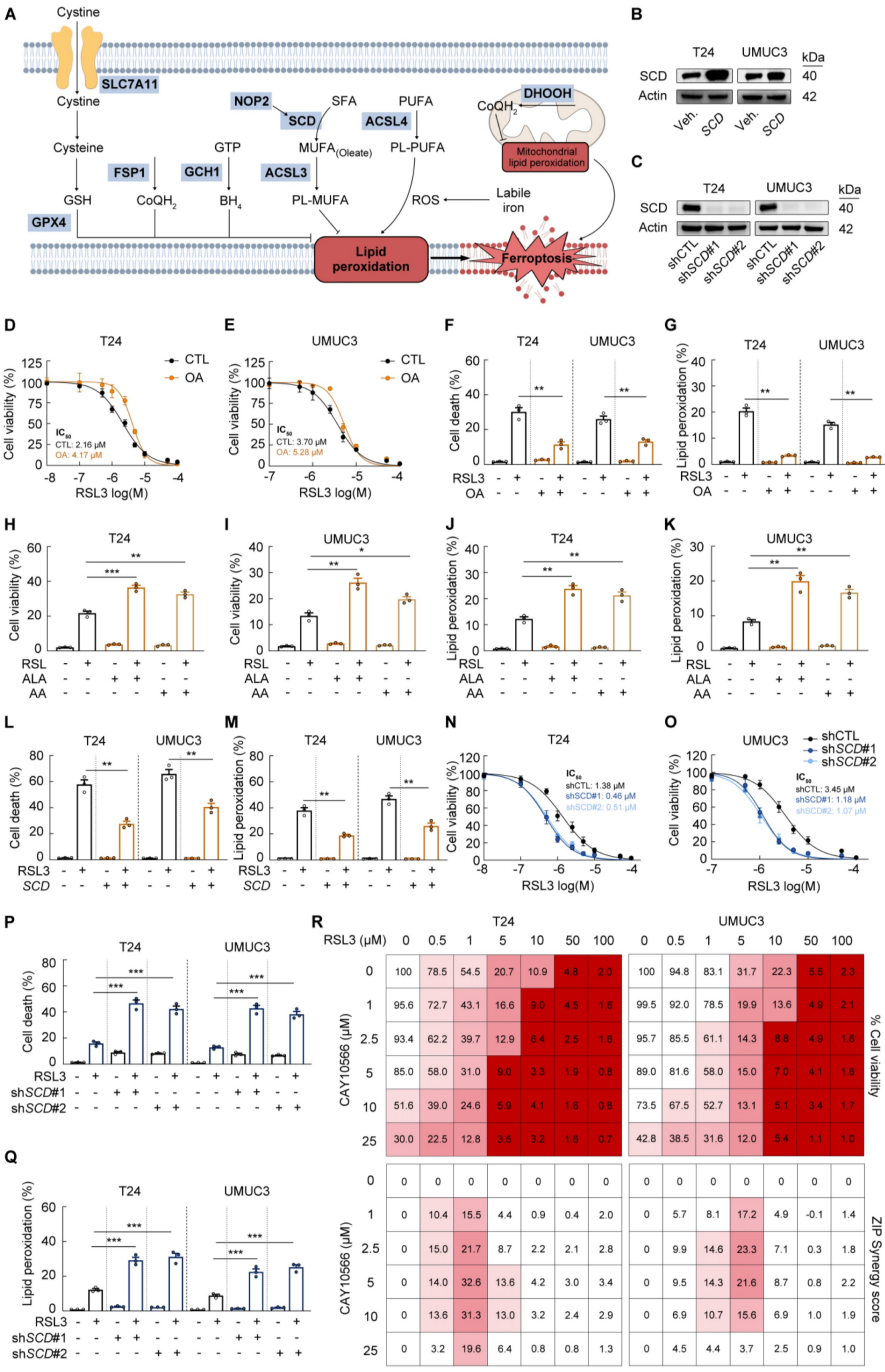
** Inhibition of SCD-dependent monounsaturated fatty acids biosynthesis sensitives BCa cells to ferroptotic cell death (A)** Schematic representation that NOP2 suppression led to decreased MUFA through SCD inhibition, resulting in increased lipid ROS formation and ferroptosis. **(B)** Western blot analysis of SCD protein levels in BCa cells following *SCD* overexpression. **(C)** Western blot analysis of SCD protein levels in BCa cells following *SCD* knockdown. **(D, E)** Cell viability assays using CCK-8 in T24 and UMUC3 cells incubated with dose-range RSL3 for 16 hours, and with vehicle (BSA) or 90 μmol/L oleate (OA). **(F, G)** Propidium iodide (PI) staining for measuring cell death **(F)**, and BODIPY 581/591 C11 staining for lipid peroxidation **(G)** in T24 and UMUC3 cells following 16-hour RSL3 treatment with or without 90 μmol/L oleate co-incubation. **(H-K)** Propidium iodide (PI) staining for measuring cell death **(H, I)**, and BODIPY 581/591 C11 staining for lipid peroxidation **(J, K)** in T24 and UMUC3 cells incubated with vehicle (BSA), 50 μmol/L α-linolenic acid (ALA), or arachidonic acid (AA) for 14 hours.** (L, M)** Propidium iodide (PI) staining for measuring cell death **(L)**, and BODIPY 581/591 C11 staining for lipid peroxidation **(M)** in T24 and UMUC3 cells with *SCD* overexpression following 16-hour RSL3 treatment. **(N, O)** Cell viability assays using CCK-8 in *SCD*-knockdown T24 and UMUC3 cells incubated with dose-range RSL3 for 16 hours. **(P, Q)** Propidium iodide (PI) staining for measuring cell death **(P)**, and BODIPY 581/591 C11 staining for lipid peroxidation **(Q)** in *SCD*-knockdown T24 and UMUC3 cells following 16-hour RSL3 treatment. **(R)** Cell viability assays using CCK-8 in T24 and UMUC3 cells incubated with dose-range RSL3 combined with CAY10566 for 12 hours. And synergy maps were determined using zero interaction potency (ZIP) synergy scores. Statistical analysis was performed using a one-way ANOVA, followed by Dunnett's test **(F-M, P-Q)**. Data are presented as means ± standard deviation; *p < 0.05, **p < 0.01, ***p < 0.001, n.s. non-significant.

Based on our findings that *NOP2* knockdown elevated cytotoxic PUFAs while decreasing protective MUFAs, we investigated whether disruption of the NOP2/SCD axis impairs lipid homeostasis and sensitizes BCa cells to ferroptosis (Fig. 5A). The GPX4 inhibitor RSL3, which blocks the enzyme's ability to utilize reduced glutathione (GSH) to detoxify lipid peroxides, exhibited antitumor activity with IC_50_ values of 1.303 μM and 3.371 μM in T24 and UMUC3 cell lines, while this cytotoxicity could be rescued by the antioxidant ferrostatin-1 (Fer-1; Supplementary Fig. S5A, B). Based on the RSL3 dose-response curves, we employed distinct RSL3 concentration ranges to attenuate or potentiate pharmacological effects in T24 and UMUC3 cells, respectively.

To determine whether the elevated PUFA/MUFA ratio enhances membrane lipid peroxidation and drives ferroptotic cell death, we experimentally modulated this ratio via direct supplementation with FAs, including oleate (OA), α-linolenic acid (ALA), and arachidonic acid (AA). MUFA supplementation increased cellular resistance to ferroptotic stress, whereas elevated PUFA levels sensitized cells to ferroptosis (Fig. 5D-K; Supplementary Fig. S5C-D). These findings mechanistically link NOP2/SCD axis dysfunction to ferroptotic vulnerability in BCa cells.

As expected, RSL3-induced cell death, lipid peroxidation, and ROS were partially reversed in SCD-overexpressing BCa cells with enhanced endogenous MUFA synthesis (Fig. 5L-M; Supplementary Fig. S5E). Conversely, SCD depletion significantly increased RSL3-induced cell death, correlating with elevated lipid peroxidation and ROS (Fig. 5N-Q; Supplementary Fig. S5F). To avoid off-target effects, we employed two SCD inhibitors, CAY10566 and MF438. Consistent with SCD depletion, both inhibitors amplified RSL3-induced cell death and lipid ROS (Supplementary Fig. S5G-L). Moreover, SCD inhibitors exhibited moderate single-agent antitumor activity and synergistic effects when combined with RSL3 in BCa cells (Fig. 5R). Collectively, these results demonstrate that the PUFA/MUFA ratio directly dictates BCa cell susceptibility to ferroptosis.

### SCD links *NOP2* knockdown and elevated ferroptosis susceptibility in bladder cancer

**Figure 6:**
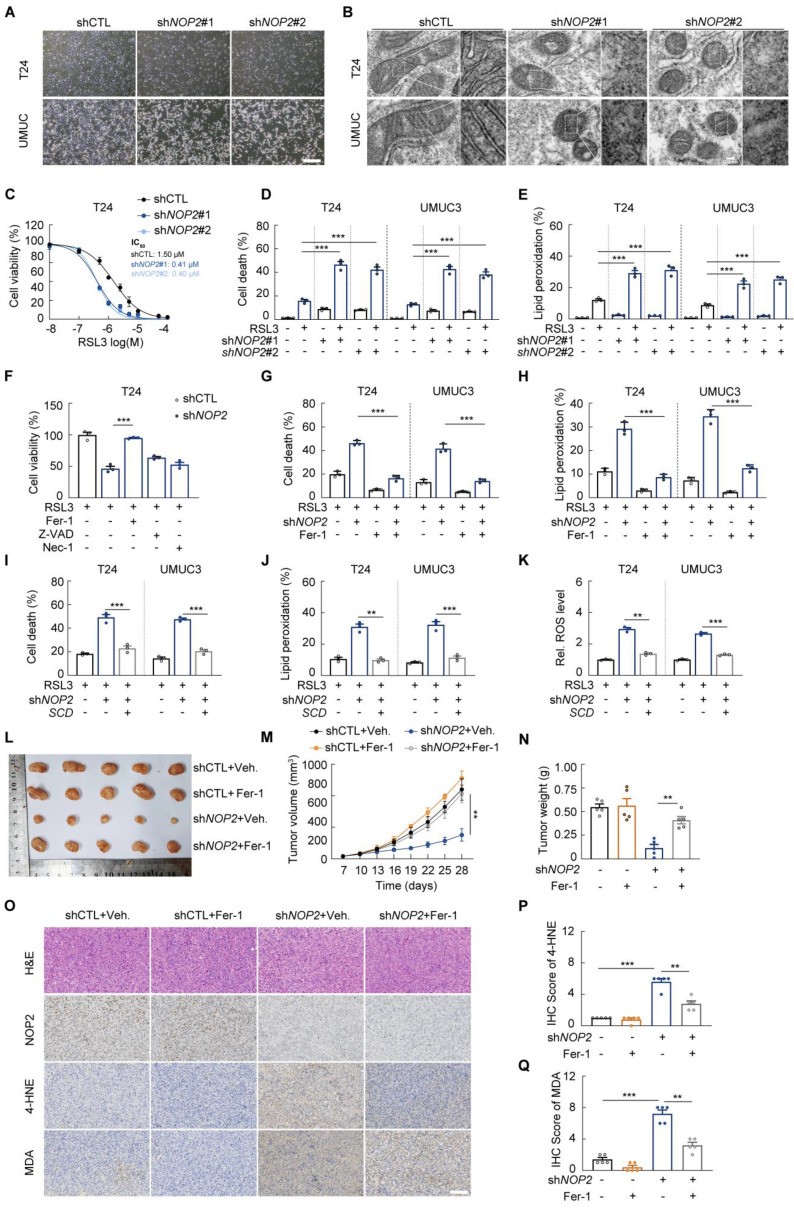
** SCD links *NOP2* knockdown and elevated ferroptosis susceptibility in bladder cancer (A)** Representative image of T24 and UMUC3 cells treated with RSL3 for 16 h. **(B)** Transmission electron microscopy images showing mitochondrial crests in T24 and UMUC3 cells treated with RSL3 for 16 h. **(C)** Cell viability assays using CCK-8 in *NOP2*-knockdown T24 cells incubated with dose-range RSL3 for 16 hours. **(D, E)** Propidium iodide (PI) staining for measuring cell death **(D)**, and BODIPY 581/591 C11 staining for lipid peroxidation **(E)** in *NOP2*-knockdown T24 and UMUC3 cells following 16-hour RSL3 treatment. **(F)** Cell viability assays using CCK-8 in BCa cells treated with or without RSL3 (1 μM), ferroptosis inhibitor Fer-1 (10 μM), apoptosis inhibitor Z-VAD-FMK (50 μM), necrosis inhibitor necrostatin-1 (Nec-1, 50 μM) as indicated for 12 hours. (G, H) Propidium iodide (PI) staining for measuring cell death **(G)**, and BODIPY 581/591 C11 staining for lipid peroxidation **(H)** in *NOP2*-knockdown T24 and UMUC3 cells following 16-hour RSL3 treatment with or without 10 μM Fer-1 co-incubation. **(I-K)** Propidium iodide (PI) staining for measuring cell death **(I)**, BODIPY 581/591 C11 staining for lipid peroxidation **(J)**, and DCFH-DA staining for ROS **(K)** in T24 and UMUC3 cells treated with indicated plasmids following 16-hour RSL3 treatment. (L-Q) Xenograft tumors of T24 or NOP2-knockdown T24 cells in BALB/c nude mice, with or without Fer-1 treatment **(L)**. Growth kinetics of the formed tumors were monitored and examined **(M)**. Tumor weights of the isolated tumor were analyzed **(N)**. NOP2, 4-HNE, and MDA staining in indicated xenograft tumors **(O)**, and immunoreactive scores of 4-HNE, and MDA **(P, Q)**. Statistical analysis was performed using a one-way ANOVA, followed by Dunnett's test **(D-K, N-Q)**, repeated measures ANOVA followed by Mauchly's test of sphericity **(M)**. Data are presented as means ± standard deviation; *p < 0.05, **p < 0.01, ***p < 0.001, n.s. non-significant.

Consequently, we investigated whether NOP2 depletion sensitizes BCa cells to ferroptosis. *NOP2*-knockdown BCa cells treated with RSL3 exhibited pronounced ferroptotic morphological features, including membrane blistering and loss of mitochondrial cristae (Fig. 6A, B) 33. The combination of NOP2 depletion and RSL3 treatment synergistically increased cell death, lipid peroxidation, and cellular ROS in T24 and UMUC3 cells (Fig. 6C-E; Supplementary Fig. S6A-B). Notably, RSL3-induced cytotoxicity in NOP2-knockdown cells was effectively rescued by co-treatment with ferroptosis inhibitors ferrostatin-1 (Fer-1) or deferoxamine (DFO) (Fig. 6F-H; Supplementary Fig. S6C-E), whereas apoptosis (Z-VAD-FMK) and necroptosis (Necrostatin-1) inhibitors showed minimal protection (Fig. 6F). These results confirm that NOP2 depletion confers ferroptosis sensitivity in BCa cells.

Moreover, we found that NOP2 dysregulation did not significantly affect either LPCAT3, ACSL4, DHODH, FSP1, GCH1 or xCT expression or intracellular Fe^2+^ content (Supplementary Fig. S6F-G). However, consistent with previous findings 34, we observed a downregulation of GPX4 protein, indicating that NOP2 loss may create a primed state for ferroptosis.

Importantly, we found that SCD ectopic expression significantly rescued ferroptosis resistance weaken by *NOP2* knockdown (Fig. 6I-K). These findings collectively demonstrate that NOP2 depletion may sensitize BCa cells to ferroptosis in an SCD-dependent manner, providing further support for the crucial role of NOP2 in ferroptosis suppression.

Given the results that *NOP2* knockdown contributes to ferroptotic cell death, we further validated whether NOP2 regulates tumor development by promoting ferroptosis evasion. We observed Fer-1 treatment rescued tumor proliferation in *NOP2*-knockdown BCa cells (Supplementary Fig. S6H-J). *In vivo* xenograft studies using T24 cells demonstrated that *NOP2* knockdown significantly inhibited tumor growth, while Fer-1 treatment effectively reversed this suppression (Fig. 6L-N). The knockdown of *NOP2* xenografts showed enhanced lipid peroxidation stained by 4-HNE and MDA, while the effect was substantially attenuated by Fer-1 treatment (Fig. 6O-Q), indicating that NOP2 is essential for promoting BCa development by suppressing ferroptosis.

### NOP2/SCD Axis suppression attenuates oncogenic activity and sensitives cells to cisplatin in bladder cancer

**Figure 7:**
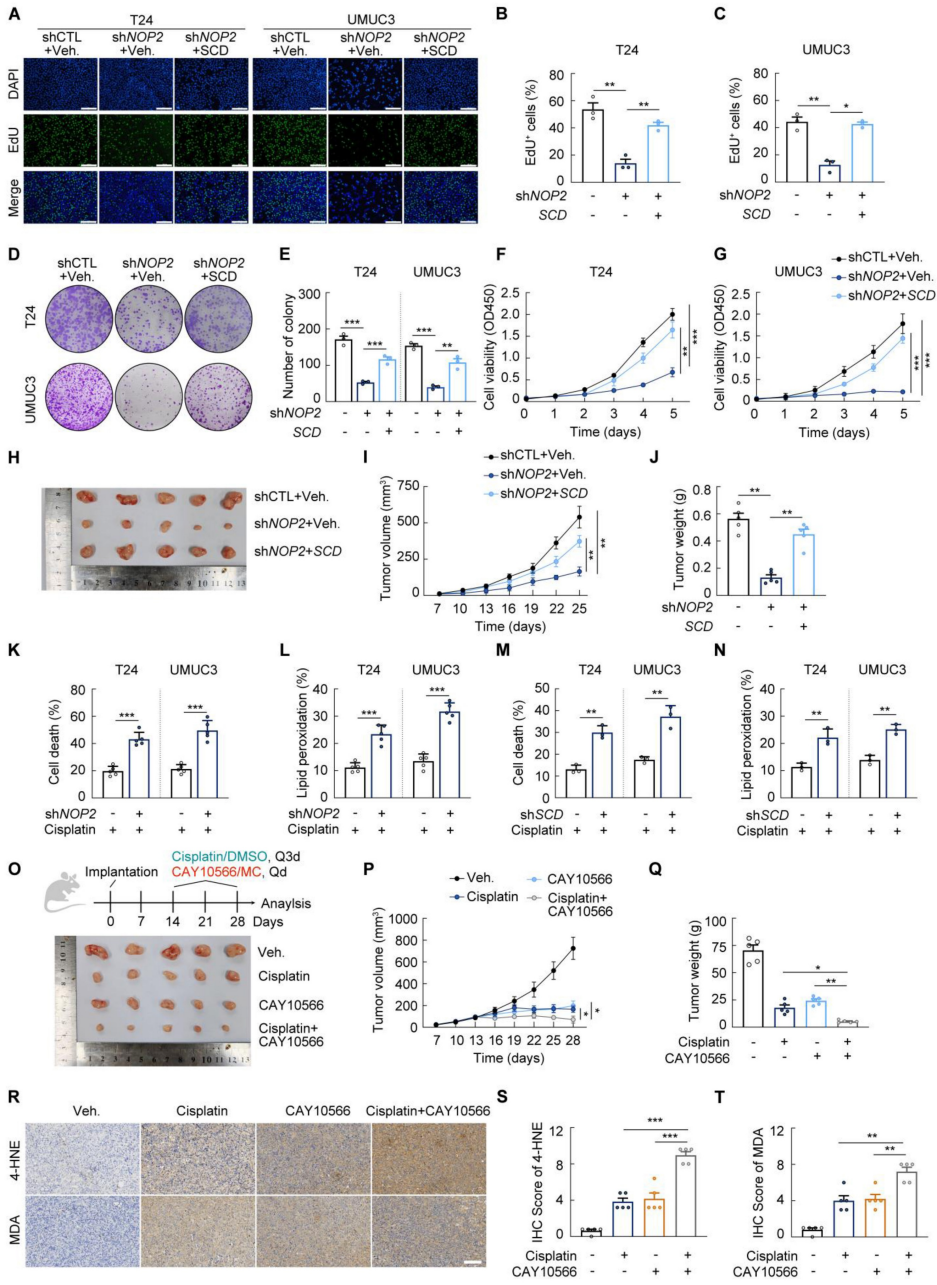
** NOP2/SCD Axis suppression attenuates oncogenic activity and sensitives cells to cisplatin in bladder cancer (A-C)** Edu assay in T24 and UMUC3 cells following the treatment with indicated vectors **(A)**, and the quantification of Edu^+^ cells were shown in **(B, C)**. **(D, E)** Colony formation assay of T24 and UMUC3 cells following the treatment with indicated vectors **(D)**, and the quantification of clones was shown in **(E)**. **(F, G)** CCK-8 assay in T24 and UMUC3 cells following the treatment with indicated vectors. **(H-J)** Xenograft tumors of T24 following the treatment with indicated vectors in BALB/c nude mice **(H)**. Growth kinetics of the formed tumors were monitored and examined **(I)**. Tumor weights of the isolated tumor were analyzed **(J)**. **(K-N)** Propidium iodide (PI) staining for measuring cell death **(K, M)**, BODIPY 581/591 C11 staining for Lipid Peroxidation **(L, N)** in *NOP2*- or *SCD-*knockdown T24 and UMUC3 cells following 24-hour cisplatin treatment. **(O-T)** Xenograft tumors of T24 cells in BALB/c nude mice, with or without cisplatin or CAY10566 treatment **(O)**. Growth kinetics of the formed tumors were monitored and examined **(P)**. Tumor weights of the isolated tumor were analyzed **(Q)**. 4-HNE, and MDA staining in indicated xenograft tumors **(R)**, and immunoreactive scores of 4-HNE, and MDA **(S, T)**. Statistical analysis was performed using a one-way ANOVA, followed by Dunnett's test **(B-C, E, J-N, Q-T)**, repeated measures ANOVA followed by Mauchly's test of sphericity **(F-G, I, P)**. Data are presented as means ± standard deviation; *p < 0.05, **p < 0.01, ***p < 0.001, n.s. non-significant.

We then investigated into the role of NOP2/SCD axis in BCa development. In rescue experiments, ectopic expression of SCD reversed the suppressive effects of *NOP2* knockdown on proliferation and invasion capacity of BCa cells *in vitro* (Figure 7A-G; Supplementary Fig. S7A-F). Consistently, ectopic expression of SCD partially restored tumor growth in subcutaneous xenografts models (Fig. 7H-J). Collectively, these results identify SCD as a key downstream effector through which NOP2-dependent m^5^C methylation promotes BCa progression.

In addition, previous studies have demonstrated that cisplatin can trigger tumor cell death by activating the ferroptosis pathway 35-37 (Supplementary Fig. S7G). We first validated that the ferroptosis inhibitor Fer-1 partly protected BCa cells from cisplatin induced cell death (Supplementary Fig. S7B-C). As expected, *NOP2* knockdown enhanced sensitivity to cisplatin of T24 and UMUC3 cell lines (Supplementary Fig. S7H-I). The cell death, lipid peroxidation and cellular ROS induced by cisplatin was increased following *NOP2* knockdown (Fig. 7K-L; Supplementary Fig. S7J-L). These results suggested suppression of NOP2 could enhance cisplatin sensitivity in BCa cells.

Given the finding that SCD is an important downstream target of NOP2, we further investigated the effect of SCD inhibition on cisplatin sensitivity in BCa. As expected, knockdown of *SCD* replicated the phenomenon of NOP2 suppression (Fig. 7M-N; Supplementary Fig. S7M). Moreover, the SCD inhibitor CAY10566 also significantly increased cisplatin-induced cellular toxicity (Supplementary Fig. S7N). To evaluate the effect of SCD suppression on cisplatin response *in vivo*, we established a therapeutic model in xenograft-bearing mice. (Fig. 7O). We observed that SCD inhibition significantly inhibited tumor growth and enhanced therapeutic sensitivity to cisplatin (Fig. 7P-Q). Moreover, the combination of cisplatin and CAY10566 showed enhanced lipid peroxidation (Fig. 7R-T). Together, these results indicated a potential strategy for enhancing sensitivity to cisplatin.

## Discussion

The dysregulation of epitranscriptomic is broadly implicated in diverse disease pathogenesis, and altered m^5^C levels have been a common feature in many cancers 8, 13, 38-43. Consistently, our findings reveal a global increase of m^5^C content in bladder cancer, paralleling the overexpression of multiple m^5^C methyltransferases. While NOP2/Sun RNA methyltransferase 2 (NSUN2) is a well-established m^5^C "writer" with oncogenic roles across cancers 22, our results position NOP2 as a key regulator that promotes BCa progression and links epitranscriptomic remodeling, metabolic reprogramming and ferroptosis evasion.

Our study demonstrates that NOP2 is significantly overexpressed in BCa patients, as evidenced by both public databases and our cohort data. This elevated expression correlates with aggressive clinicopathological features, including greater tumor burden, higher grade, and advanced disease stage, suggesting its clinical relevance. Mechanistically, NOP2-mediated m^5^C deposition in the *SCD* mRNA 3'-UTR recruits the m^5^C “reader” protein YBX1, thus stabilizing *SCD* mRNA and boosting the expression of SCD. Upregulated SCD expression, in turn, increases the incorporation of MUFAs into membrane phospholipids to inhibit lipid peroxidation and protect BCa cells from ferroptotic cell death. Moreover, this protective effect was abolished by a catalytic inactive NOP2 mutant (NOP2^C513A^), confirming the m^5^C-dependent specificity of the mechanism. The elucidation of underlying biological mechanism highlights the potential of NOP2 as a biomarker for risk stratification and a target for therapy.

The identification of NOP2 as a key regulator of BCa progression broadens the functional scope of RNA modifications in urinary malignancies. Beyond confirming that NOP2-mediated m^5^C methylation actively sustains SCD expression to promote cancer cell survival which aligns the existing evidences that RNA modifications regulate tumor progression 18, our study moves beyond prior work by revealing ferroptosis evasion as a direct and defined consequence of epitranscriptomic reprogramming. Furthermore, the critical dependence of the novel NOP2/SCD axis on m^5^C-binding protein YBX1 highlights the importance of RNA-protein interactions and dynamic of this regulatory axis in BCa biology. These findings integrate m^5^C-based epitranscriptomic mechanisms into the molecular landscape of urinary malignancy, providing a broader perspective for understanding its complex pathogenesis.

NOP2-mediated epitranscriptomic regulation may prime ferroptosis through a mechanism independent of known pathways involving ACSL4, LPCAT3, FSP1, GCH1, and DHODH 44. This effect is likely mediated by SCD, a key anti-ferroptotic lipid metabolism enzyme 14, 15, 34. By sustaining SCD expression, NOP2 drives a lipid remodeling program that enriches membrane phospholipids with MUFAs while depleting peroxidation-susceptible PUFAs. This shift in membrane composition, in turn, maintains membrane integrity and protects cells against ferroptosis. Notably, recent studies have redefined lipid droplets from static lipid storage depots to dynamic, multifunctional organelles that actively respond to cellular stress 45-47. For instance, upon acute oxidative damage induced by high-dose H₂O₂, lipid droplets can sequester toxic, lipophilic components released from damaged mitochondria, thereby functioning as a cytoprotective sink to mitigate lipid toxicity and membrane damage 45. A dual-targeting polarity-sensitive ratiometric fluorescence probe has revealed a significant increase in lipid droplet polarity and a gradual homogenization of polarity between lipid droplets and the cytoplasm during ferroptosis, providing direct visual evidence of lipid droplet remodeling under oxidative lipid stress 46. Another research has developed Ru(II) lipid-mimics inducing neutral lipids phase separation, increasing PUFAs, rendering cells more sensitive to ferroptosis and eliciting robust immune responses 47. Collectively, these findings suggests that the cellular capacity to buffer oxidative stress is not solely determined by the composition of membrane phospholipids, but also by the functionality of lipid droplets, suggesting that NOP2/SCD axis likely regulate cellular sensitivity to ferroptosis through multiple mechanisms.

While NOP2 or SCD depletion alone does not induce significant lipid peroxidation or cellular death under baseline conditions, it elevated peroxidation trends and decreased GPX4 levels, indicating a lower ferroptotic threshold and lower cellular adaptivity. This state sensitizes BCa cells to ferroptosis inducer such as RSL3, supporting a double hit model where epitranscriptomic dysregulation primes the cell for ferroptotic cell death execution by a secondary stressor. Consistent with this finding, *NOP2* knockdown or the SCD inhibitor CAY10566 treatment synergized with RSL3 to enhance BCa cell death *in vitro*. Thus, this decoupled ferroptosis initiation highlights the importance of NOP2-SCD in cellular redox homeostasis.

This ferroptosis-primed state represents a novel therapeutic vulnerability. Standard chemotherapy agent cisplatin is found to generate ROS stress 35-37, which can serve as a trigger for ferroptosis. In the absence of a direct NOP2 inhibitor, we therefore utilized SCD inhibitor CAY10566. We found Pharmacological inhibition of SCD synergized strongly with cisplatin. These work highlights the translational promise of SCD inhibition and justifies the development of NOP2-targeted therapies.

While our study establishes a novel oncogenic role for the NOP2/YBX1/SCD axis in bladder cancer progression, it is important to acknowledge certain limitations. The primary *in vivo* evidence presented here relies on a subcutaneous xenograft model. Although this model robustly confirmed the tumor-promoting function of NOP2, it does not fully recapitulate the complex tumor microenvironment (TME) of the native bladder, which includes interactions with the urothelial stroma, immune cells, and unique physiological pressures.

In summary, our study establishes the oncogenic role of the m^5^C methyltransferase NOP2 in bladder cancer. We demonstrate that NOP2-mediated m^5^C modification on *SCD* mRNA orchestrates lipid metabolic reprogramming and thereby suppressing ferroptosis and promoting tumor progression.

## Supplementary Material

Supplementary methods, figures and tables 1-4, 6.

Supplementary table 5.

## Data Availability

Further information and requests for resources and reagents should be directed to and will be fulfilled by the Lead Contact, Guanghou Fu (fuguanghou@zju.edu.cn).

## Abbreviations

NOP2: NOP2/Sun RNA methyltransferase 1
m^5^C: 5-methylcytosine
YBX1: Y-Box Binding Protein 1
SCD: Stearoyl-CoA Desaturase
BCa: bladder cancer
TCGA: The Cancer Genome Atlas
siRNA: Small interfering RNA
shRNAs: Short hairpin RNA
DEG: Differentially expressed gene
KEGG: Kyoto Encyclopedia of Genes and Genomes
GSEA: Gene Set Enrichment Analysis
RNA-Bis-Seq: RNA Bisulfite Sequencing
WT: Wild type
MUT: Mutant
PI: Propidium iodide
SFA: Saturated fatty acid
MUFA: Monounsaturated fatty acid
PUFA: Polyunsaturated fatty acid
FADS: Fatty Acid Desaturase 1
ELOVL: Elongation Of Very Long Chain Fatty Acids
FA: fatty acid
PL: Phospholipids
PC: phosphatidylcholine
PE: phosphatidylethanolamine
TG: triglyceride
GSH: glutathione
Fer-1: Ferrostatin-1
BSA: Bovine Serum Albumin
4-HNE: 4-Hydroxynonenal
MDA: malondialdehyde
