# Supplementary material for: NOP2-mediated 5-methylcytosine Regulates Lipid Metabolism Reprogramming to Prime Tumors for Ferroptosis in Bladder Cancer Progression
Source: Int J Biol Sci. 2026 Apr 16;22(8):4399–416. doi: 10.7150/ijbs.126325 (PMC13137962; doi:10.7150/ijbs.126325)
Supplement: Supplementary file 1 — Supplementary methods, figures and tables 1-4, 6. [file ijbsv22p4399s1.pdf]

Supplemental Fig. S1

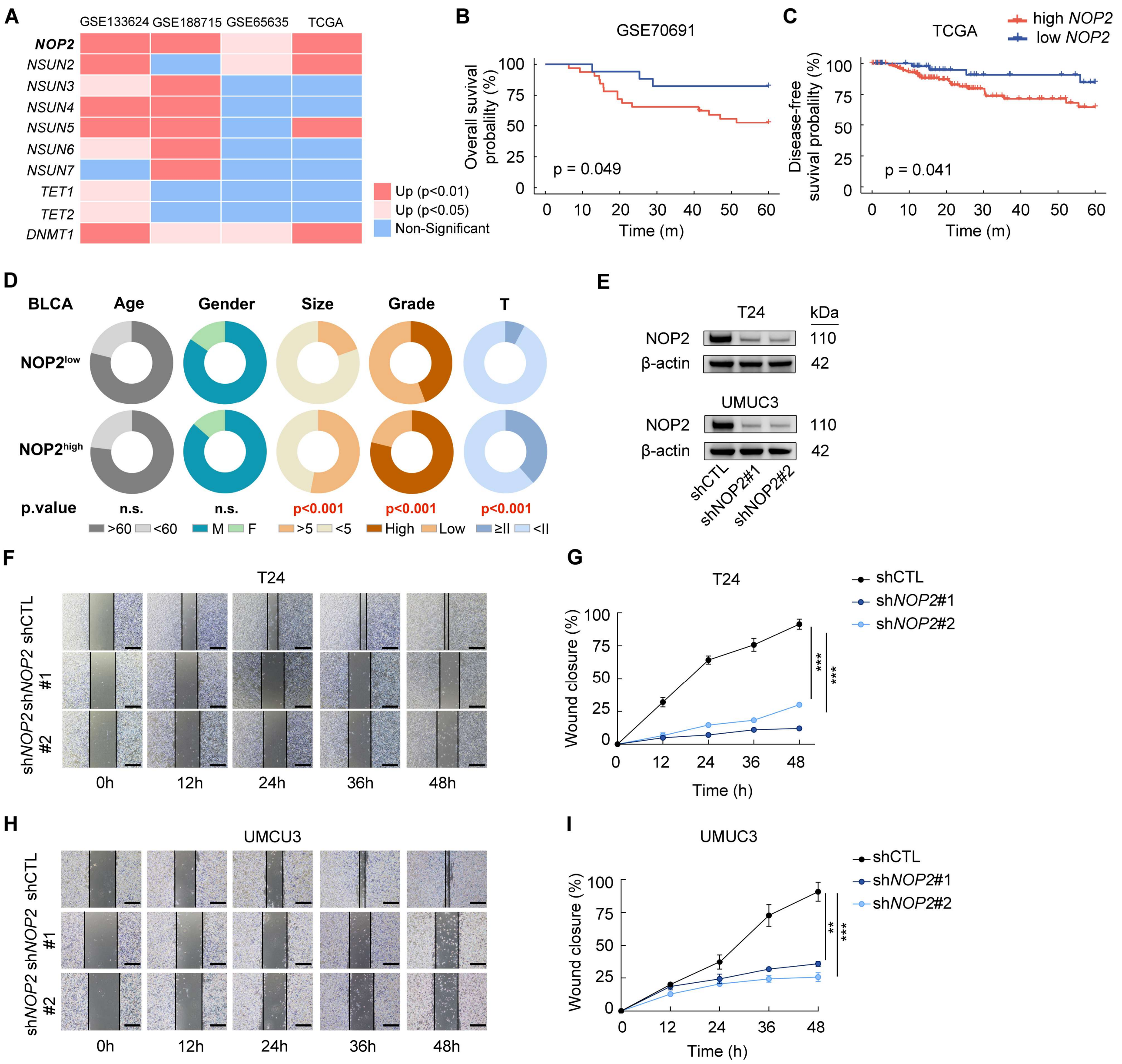

Supplementary Fig. S1 NOP2 is upregulated in BCa tissues and drives tumor progression (A) Comparison of the expression of 10 m^5C regulators between BCa tissues and paracancerous tissues from GEO and TCGA database. (B) Kaplan-Meier survival analysis of *NOP2* in BCa patients (GSE70691 cohort; n = 49). (C) Kaplan-Meier survival analysis of *NOP2* in BCa patients (TCGA-BLCC cohort; n = 186). (D) Pie charts representing the association between clinicopathologic features and NOP2 protein expression in the BCa cohort. (E) Western blot analysis of NOP2 protein levels in *NOP2*-knockdown T24 and UMUC3 cells. (F-I) Wound healing assay of *NOP2*-knockdown T24 and UMUC3 cells (F, H), and the quantification of wound closure were shown in (G, I). Statistical analysis was performed using a repeated measures ANOVA followed by Mauchly's test of sphericity (G, I). Data are presented as means ± standard deviation; $^*p < 0.05$, $^{**}p < 0.01$, $^{***}p < 0.001$, n.s. non-significant.

Supplemental Fig. S2

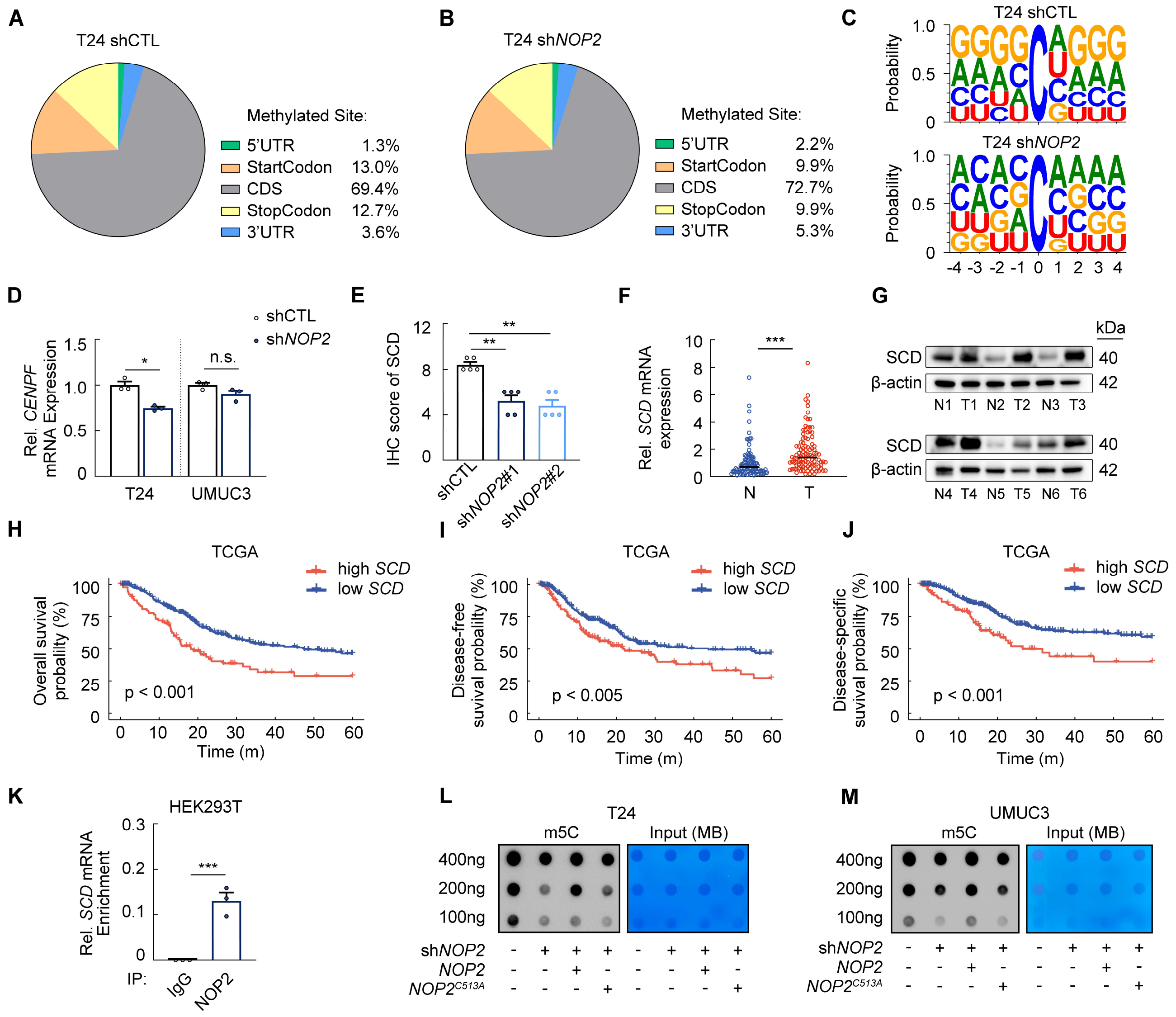

Supplementary Fig.S2 NOP2 promotes SCD expression via an m^{5}C-dependent manner in bladder cancer

(A, B) m^{5}C distributions within different regions in *NOP2* knockdown cells and the controls. (C) Top consensus m^{5}C motif in in *NOP2* knockdown cells and the controls. (D) *CENPF* mRNA levels in *NOP2*-knockdown BCa cells and the controls. (E) The immunoreactive scores quantified for SCD in xenograft tumors. (F) *SCD* mRNA levels in tumor and paracancerous tissues from BCa patients. (G) Western blot analysis of SCD protein levels in tumor and paracancerous tissues from BCa patients. (H-J) Kaplan-Meier survival analysis of *SCD* in BCa patients (TCGA-BLCA cohort; n = 405). (K) RIP-qPCR analysis of binding ability of NOP2 to *SCD* mRNA in HEK293T cells. (L, M) Dot blot analysis of the m5C modification in T24 cells following the treatment with indicated vectors. Statistical analysis was performed using a one-way ANOVA, followed by Dunnett's test (E) or unpaired two-tailed student t-test (D, F, K). Data are presented as means ± standard deviation; *p < 0.05, **p < 0.01, ***p < 0.001, n.s. non-significant.

Supplemental Fig. S3

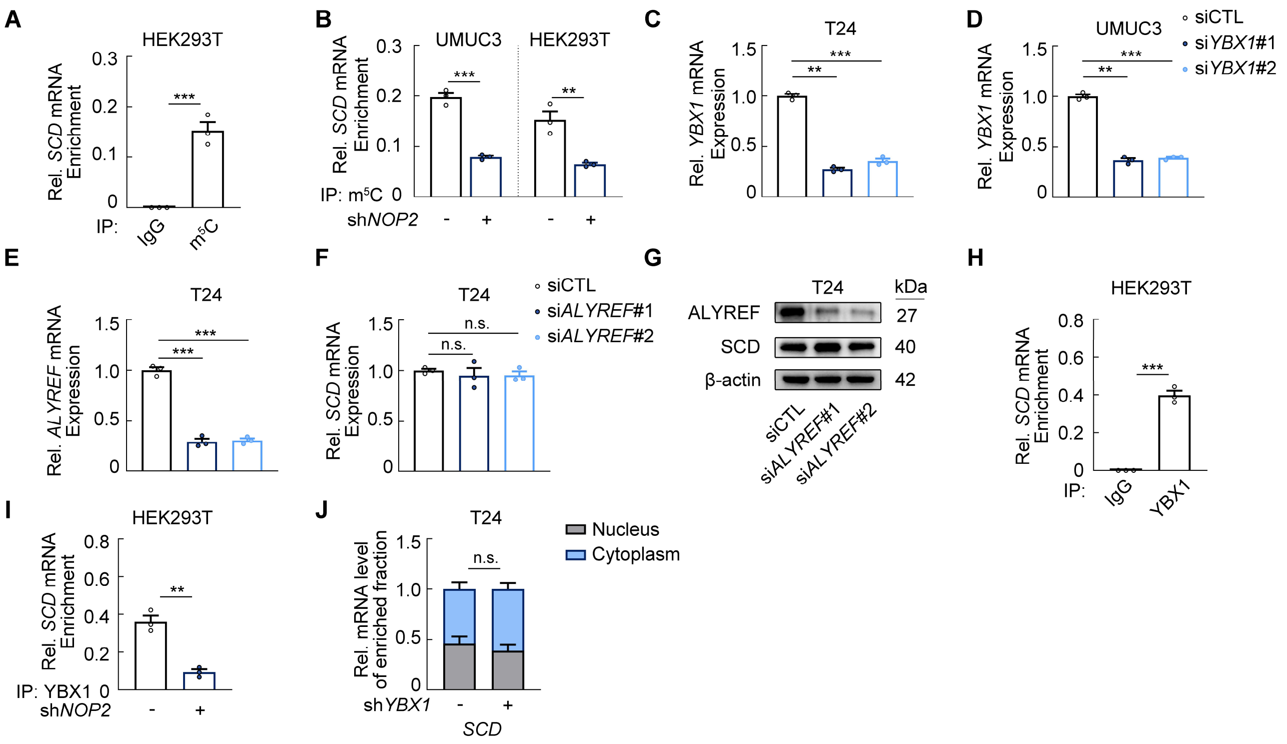

Supplementary Fig.S3 YBX1 recognizes and stabilizes m^5C modified *SCD* mRNA (A) RIP-qPCR analysis of m^5C modification of *SCD* mRNA in HEK293T cells. (B) RIP-qPCR analysis of m^5C modification upon SCD RNA in *NOP2*-knockdown UMUC3 and HEK293T cells. (C, D) *YBX1* mRNA levels in *YBX1*-knockdown BCa cells and the controls. (E) *ALYREF* mRNA levels in *ALYREF*-knockdown BCa cells and the controls. (F) *SCD* mRNA levels in *ALYREF*-knockdown BCa cells and the controls. (G) Western blot analysis of SCD protein levels in *ALYREF*-knockdown T24 cells. (H) RIP-qPCR analysis of binding ability of YBX1 to *SCD* mRNA in HEK293T cells. (I) RIP-qPCR analysis of binding ability of YBX1 to *SCD* mRNA in *NOP2*-knockdown HEK293T cells and the controls. (J) RT-qPCR analysis of SCD mRNA nucleocytoplasmic shuttling. Statistical analysis was performed using a one-way ANOVA, followed by Dunnett's test (C-F) or unpaired two-tailed student t-test (A-B, H-J). Data are presented as means ± standard deviation; *p < 0.05, **p < 0.01, ***p < 0.001, n.s. non-significant.

Supplemental Fig. S4

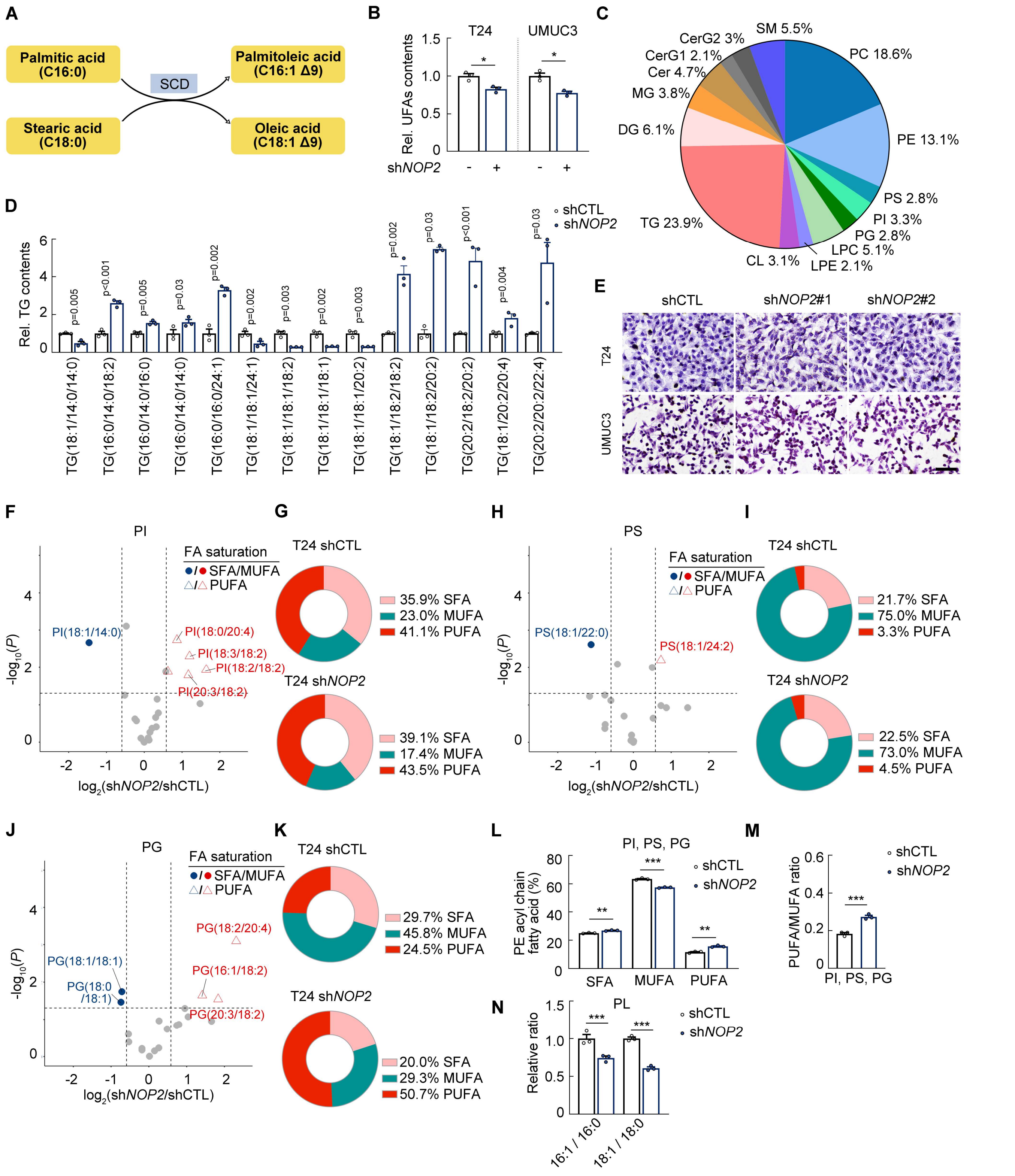

Supplementary Fig. S4 Knockdown of *NOP2* decreases monounsaturated fatty acids dependent on SCD (A) Schematic representation of biological function of SCD. (B) UFA quantification after knockdown of NOP2 in T24 and UMUC3 cells. (C) Composition of lipid classes in T24 and *NOP2*-knockdown T24 cells detected by liquid chromatography–mass spectrometry. (D) Relative abundance of triglyceride species in *NOP2* knockdown T24 and control cells. (E) Oil-red O staining in *NOP2* knockdown BCa cell lines. (F-M) Lipidomics showing the distribution

of SFAs, MUFAs, and PUFAs in PI (F, G), PS (H, I), and PG (J, K) in *NOP2* knockdown T24 cells compared with control. PUFA:MUFA ratio for PI, PS and PG are shown in (L, M). (N) Relative abundance of total PL (16:1/16:0) and PL (18:1/18:0) in *NOP2* knockdown T24 cells and the controls. Statistical analysis was performed using an unpaired two-tailed student t-test. Data are presented as means ± standard deviation; *$p < 0.05$, **$p < 0.01$, ***$p < 0.001$, n.s. non-significant.

Supplemental Fig. S5

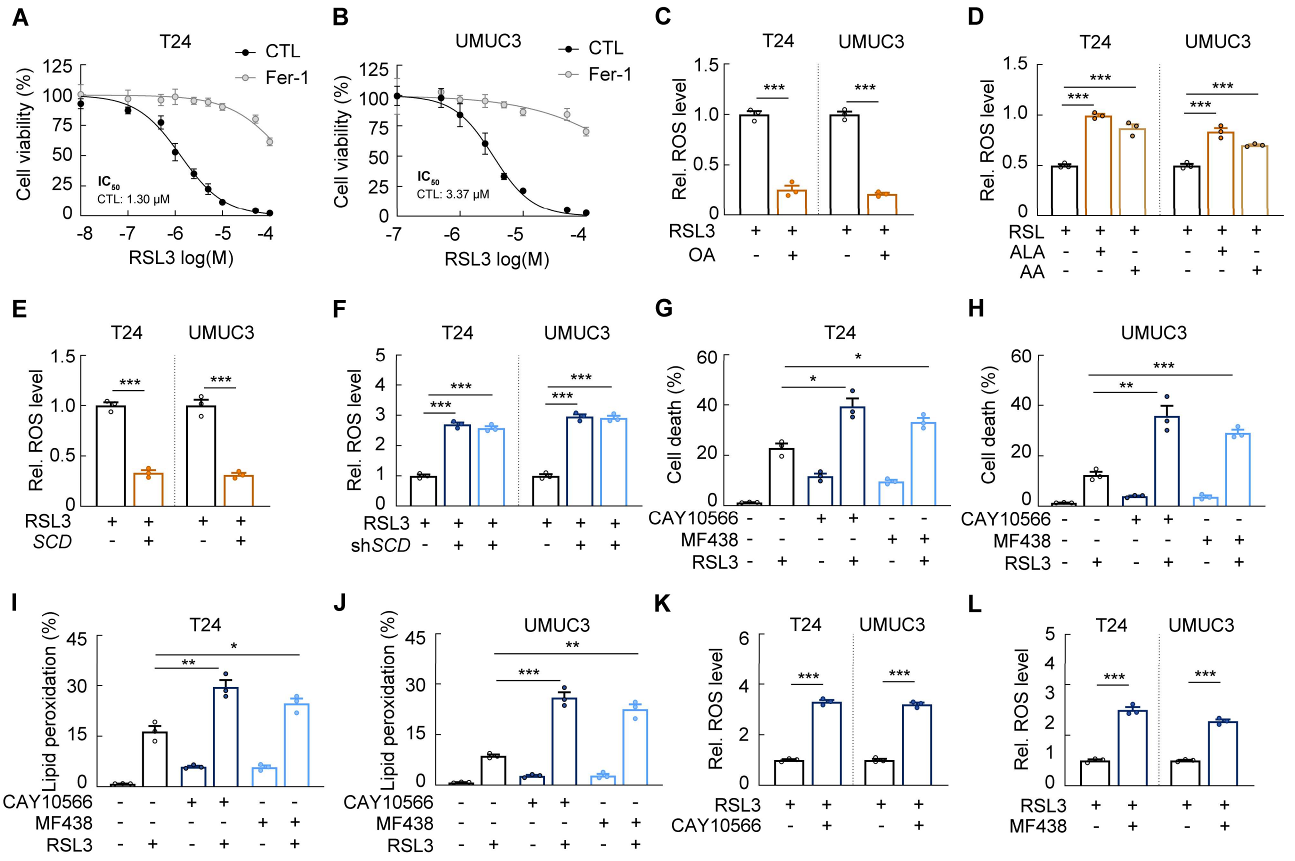

Supplemental Fig.S5 Inhibition of SCD-dependent monounsaturated fatty acids biosynthesis sensitives BCa cells to ferroptotic cell death (A, B) Cell viability assays using CCK-8 in T24 and UMUC3 cells incubated with dose-range RSL3 for 16 hours, and with vehicle or 10 μmol/L Fer-1. (C, D) DCFH-DA staining for ROS in T24 and UMUC3 cells following 16-hour treatment with RSL3 and FFAs. (E) DCFH-DA staining for ROS in T24 and UMUC3 cells with SCD overexpression following 16-hour treatment with RSL3. (F) DCFH-DA staining for ROS in *SCD*-knockdown T24 and UMUC3 cells incubated with dose-range RSL3 for 16 hours. (G-L) Propidium iodide (PI) staining for measuring cell death (G, H), BODIPY 581/591 C11 staining for lipid peroxidation (I, J), and DCFH-DA staining for ROS (K, L) in T24 and UMUC3 cells following 12-hour RSL3 treatment with 1.5 μmol/L CAY10566 or 1 μmol/L MF438 co-incubation. Statistical analysis was performed using a one-way ANOVA, followed by Dunnett's test (F-J) or unpaired two-tailed student t-test (C-E, K, L). Data are presented as means ± standard deviation; *p < 0.05, **p < 0.01, ***p < 0.001, n.s. non-significant.

Supplemental Fig. S6

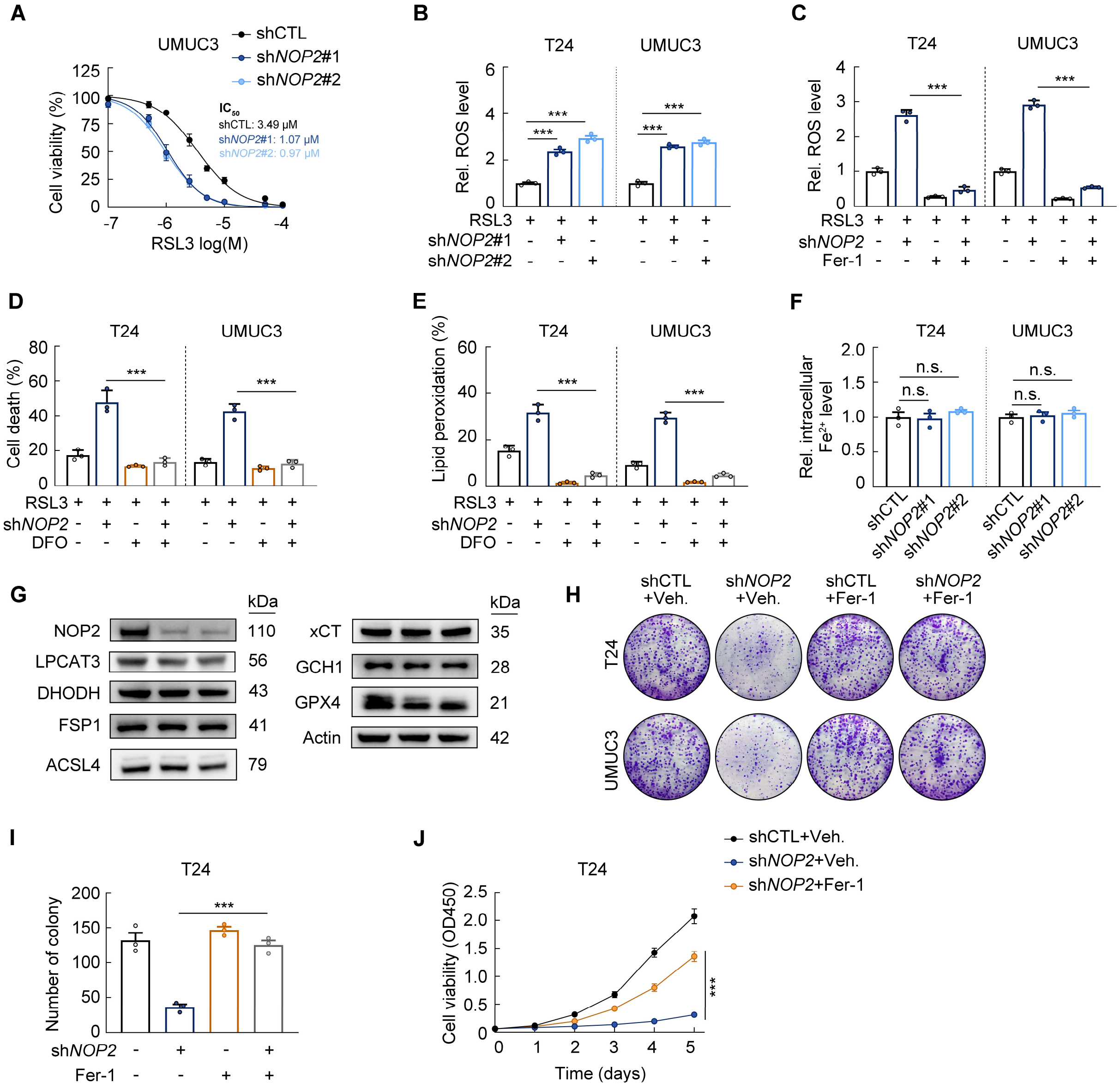

Supplementary Fig. S6 SCD links *NOP2* knockdown and elevated ferroptosis susceptibility in bladder cancer (A) Cell viability assays using CCK-8 in *NOP2*-knockdown UMUC3 cells incubated with dose-range RSL3 for 16 hours. (B) DCFH-DA staining for ROS in *NOP2*-knockdown T24 and UMUC3 cells incubated with dose-range RSL3 for 16 hours. (C) DCFH-DA staining for ROS in *NOP2*-knockdown T24 and UMUC3 cells following 16-hour RSL3 treatment with or without 10 μM Fer-1 co-incubation. (D, E) Propidium iodide (PI) staining for measuring cell death (D), and BODIPY 581/591 C11 staining for lipid peroxidation (E) in *NOP2*-knockdown T24 and UMUC3 cells following 16-hour RSL3 treatment with or without 50 μM DFO co-incubation. (F) Ferro-Orange staining analysis of intracellular Fe^{2+} levels in *NOP2*-knockdown T24 and UMUC3 cells. (G) Western blot analysis of LPCAT3, DHODH, FSP1, ACSL4, xCT, GCH1, and GPX4 expression in *NOP2*-knockdown T24 cells. (I, J) Colony formation assay of *NOP2*-knockdown T24 and UMUC3 cells with or without Fer-1 incubation (I), and the

quantification of clones were shown in (J). (K) CCK-8 assay in *NOP2*-knockdown T24 cells with or without Fer-1 incubation. Statistical analysis was performed using a one-way ANOVA, followed by Dunnett's test (B-J), repeated measures ANOVA followed by Mauchly's test of sphericity (K). Data are presented as means ± standard deviation; *p < 0.05, **p < 0.01, ***p < 0.001, n.s. non-significant.

Supplemental Fig. S7

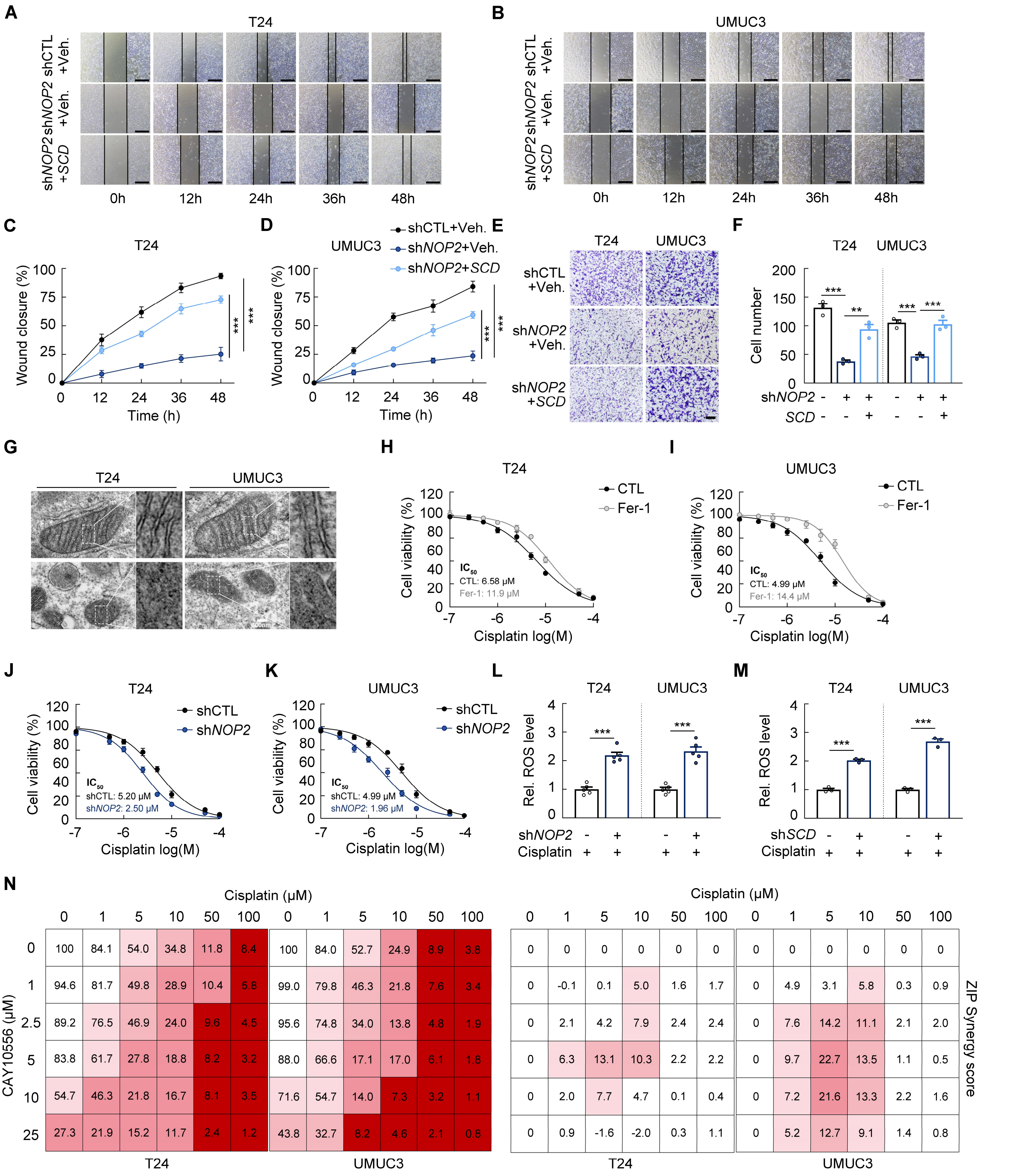

Supplementary Fig.S7 NOP2/SCD Axis suppression attenuates oncogenic activity and sensitives cells to cisplatin in bladder cancer

Supplementary Fig.S7 NOP2/SCD Axis suppression attenuates oncogenic activity and sensitives cells to cisplatin in bladder cancer (A-D) Wound healing assay of T24 and UMUC3 cells following the treatment with indicated vectors (A, B), and the quantification of wound closure were shown in (C, D). (E, F) Transwell assay of NOP2-knockdown T24 and UMUC3 cells following the treatment with indicated vectors (E), and the quantification of trans-membrane cells were shown in (F). (G) Transmission electron microscopy images showing mitochondrial

crests in T24 and UMUC3 cells treated with cisplatin for 24 h. (H, I) Cell viability assays using CCK-8 in T24 and UMUC3 cells incubated with dose-range cisplatin for 24 hours, and with vehicle or 10 μmol/L Fer-1. (J, K) Cell viability assays using CCK-8 in *NOP2*-knockdown T24 and UMUC3 cells incubated with dose-range cisplatin for 24 hours. (L, M) DCFH-DA staining for ROS in *NOP2*- or *SCD*-knockdown T24 and UMUC3 cells following 24-hour RSL3 treatment. (N) Cell viability assays using CCK-8 in T24 and UMUC3 cells incubated with dose-range cisplatin combined with CAY10566 for 24 hours. And synergy maps were determined using zero interaction potency (ZIP) synergy scores. Statistical analysis was performed using a one-way ANOVA, followed by Dunnett's test (F), repeated measures ANOVA followed by Mauchly's test of sphericity (C-D), or unpaired two-tailed student t-test (L-M). Data are presented as means ± standard deviation; *p < 0.05, **p < 0.01, ***p < 0.001, n.s. non-significant.

Supplementary Table S1. Clinicopathological characteristics in local cohort of BCa patients

Characteristics	patients (n=103)	NOP2 expression		P value
		Low	High	
Age				0.8107
<=60	22	10	12	
>60	81	41	40	
Gender				0.7866
Male	88	43	45	
Female	15	8	7	
Tumor size				0.0009
<=2.5cm^3	66	41	25	
>2.5cm^3	37	10	27	
Histologic grade				0.0005
Grage1 & 2	39	28	11	
Grade3	64	23	41	
T stage				0.001
Ta, Tis & T1	78	46	32	
T2-T4	25	5	20	

Statistical significance was determined by Mann-Whitney U test.

Supplementary Table S2. siRNA, and shRNA sequences used in this study.

siRNA/shRNA		Sequence (5'-3')
shNOP2#1	Target sequence	GCCTTCCAGAAACAGAATGAT
shNOP2#2	Target sequence	ATGAGTGGGTGGTAGACTATG
shSCD#1	Target sequence	GCACAUCAACUUCACCACA
shSCD#2	Target sequence	GGAGAAACAUCAUCCUUAUUU
siYBX1#1	Sense<br>Anti-Sense	GGCAAUGAAGAAGAUAAAGAAAATT<br>UUUUCUUUAUCUUCUUCAUUGCCTT
siYBX1#2	Sense<br>Anti-Sense	GGAGUUUGAUGUUGUUGAAGGTT<br>CCUUCAACAACAUCAAACUCCTT
siALYREF#1	Sense<br>Anti-Sense	CGUGGAGACAGGUGGGAAATT<br>UUUCCCACCUGUCUCCACGTT
siALYREF#2	Sense<br>Anti-Sense	GGAGUCUCAGACGCCGAUAUUTT<br>AAUAUCGGCGUCUGAGACUCCTT

Supplementary Table S3. Primer sequences used in this study.

Primer name	Sequence (5'-3')
F-GAPDH	GGAGCGAGATCCCTCCAAAAT
R-GAPDH	GGCTGTTGTCATACTTCTCATGG
F-NOP2	GCATTCTGTACCATGGGGCG
R-NOP2	AATCTCCTCTTGGCTGCCCT
F-SCD	CCGGACACGGTCACCCGTTG
R-SCD	TGGTCGATCGCACGTTCCGC
F-CENPF	CTCTCCCGTCAACAGCGTTC
R-CENPF	GTTGTGCATATTCTTGGCTTGC
F-YBX1	GAGAAGTGATGGAGGGTGCT
R-YBX1	TTAGGGTTTTCTGGGCGTCT
F-ALYREF	GCAGGCCAAAACAACTTCCC
R-ALYREF	AGTTCCTGAATATCGGCGTCT
MeRIP-F-SCD	CAGCTTCTAAGAGCGAACTTC
MeRIP-R-SCD	ATTTTATTAAGACTCCCAATAACTCACTC

Supplementary Table S4. Probes used for RNA pulldown in this study.

probe name	sequence (5'-3')
SCD [m5C]	AGGAAGGGATCTGAGAACACGTTGCC[m^{5}C]AGGGGCTTGAG AAGGTTACTGAG
SCD [C]	AGGAAGGGATCTGAGAACACGTTGCCAGGGGCTTGAG AAGGTTACTGAG

Supplementary Table S6 The antibodies and inhibitors used in this study.

Antibody	Cat. #	Supplier
NOP2 Rabbit Monoclonal antibody	ab271075	abcam
SCD Rabbit Polyclonal antibody	A16429	ABclonal
5-Methylcytosine (5-mC) Rabbit Monoclonal antibody	ab214727	abcam
YBX1 Rabbit Monoclonal antibody	ab76149	abcam
ALYREF Rabbit Monoclonal antibody	ab202894	abcam
xCT/SLC7A11 Rabbit Monoclonal antibody	12691	Cell Signaling Technology
GPX4 Rabbit Monoclonal antibody	52455	Cell Signaling Technology
AIFM2/FSP1 Rabbit Monoclonal antibody	51676	Cell Signaling Technology
DHODH Rabbit Monoclonal antibody	ab174288	abcam
GCH1 Polyclonal antibody	28501-1-AP	Proteintech
Ki-67 Polyclonal antibody	27309-1-AP	Proteintech
4-Hydroxynonenal Antibody	MAB3249-SP	Novus Biologicals
Malondialdehyde Antibody	NBP2-59367	Novus Biologicals
β-actin Rabbit Monoclonal antibody	66009-1-Ig	Proteintech
HRP-conjugated Goat Anti-Rabbit IgG(H+L)	SA00001-2	Proteintech

Methods

Clinical specimens

This study was conducted in accordance with medical ethics guidelines and was approved by the Institutional Ethics Committee of the First Affiliated Hospital, Zhejiang University School of Medicine (IIT20210265B-R1). A total of 103 pairs of bladder cancer (BCa) and matched adjacent normal tissues were procured from the same institution between January 2022 and January 2024. Informed consent was obtained from the patients. The clinicopathological characteristics of the cohort are summarized in Supplementary Table S1. Additionally, transcriptomic data and corresponding clinical information for the TCGA-BLCA cohort were retrieved from the TCGA data portal (https://portal.gdc.cancer.gov).

Cell culture

The human embryonic kidney 293 T cells (HEK293T) cell line and two human BCa cell lines, T24 and UMUC3, were acquired from the Chinese Academy of Science (Shanghai, China). All cells were cultured in required medium supplied with 10% Fetal Bovine Serum (FBS; Gibco, cat. #A5256701) and 1% Penicillin/Streptomycin (Procell, cat. #PB180120) at 37°C in a 5 % CO_2 atmosphere.

Animal studies

All animal procedures were conducted in an accordance with guidelines approved by the Institutional Ethics Committee of the First Affiliated Hospital, Zhejiang University School of Medicine. Mice were purchased from Zhejiang Animal Center and housed under specific-pathogen-free facility conditions. For xenograft tumor formation, 5×10^6 T24 cells suspended in Matrigel (MCE; cat. #HY-K6001) were subcutaneously (s.c.) injected into 6-week-old male BALB/c nude mice. Ferrostatin-1 (Fer-1; MCE, cat. #HY-100579) was administered intraperitoneally (i.p.) at a dose of 5 mg/kg every 3 days for 2 weeks. The dose and dosing schedule used with CAY10566 (MCE, cat. #HY-15823), cisplatin (MCE, cat. #HY-17394) or vehicle controls was 5 mg/kg administered i.p. at 3-4day intervals. Tumor dimensions were measured using caliper, and tumor volume was determined based on the formula: V = (Length × Width × Height)/2.

RNA interference, lentiviral infection and plasmid transfection

Small interfering RNAs (siRNAs; sequence listed in Supplementary Table S2) were synthesized by SUNYA Company (Hangzhou, China). Lentiviral vectors were generated by GeneChem (Shanghai, China). Short hairpin RNAs (shRNAs; target sequence listed in Supplementary Table S2) were cloned into the GV492 vector. Lentiviral infection was carried out following established protocols [13]. Overexpression plasmids were obtained from

Transheep Company (Shanghai, China). Mutations in the NOP2 gene were introduced using overlap extension PCR. Transfection of siRNAs or plasmids was performed using Lipofectamine 3000 (Invitrogen, cat. #L3000015) according to the manufacturer's instructions.

RNA extraction and RT-qPCR

Total RNA was extracted from cultured cells and tissue samples using TRIzol reagent (Life Technologies, Austin, USA). cDNA was synthesized using the HiScript III RT SuperMix for qPCR (+gDNA wiper) kit (Vazyme, cat. #R323-01) according to the manufacturer's protocol. Quantitative polymerase chain reaction (qPCR) was then carried out with ChamQ Universal SYBR qPCR Master Mix (Vazyme, cat. #Q711-02). Gene expression levels were quantified via the $2^{\wedge-\Delta\Delta}$CT method, with GAPDH mRNA serving as the internal normalization control. The sequences of all primers used are provided in Supplementary Table S3.

Western blot

Western blot analysis was performed according to previously described methods [13]. Primary and secondary antibodies used are listed in Supplementary Table S6. Protein bands were visualized using an enhanced chemiluminescence detection system (Beyotime, cat. #P0018S).

Dot blot

Total RNA was extracted from treated cells and resuspended in three volumes of RNA incubation buffer, followed by denaturation at 65 °C for 5 minutes. Different amounts of RNA (400, 200, and 100ng) were applied to Amersham Hybond N^+ membranes (GE Healthcare, USA) using a Bio-Dot apparatus (Bio-Rad, USA) with a mixture of 20 × SSC buffer (pre-cooled at 4°C; Sigma-Aldrich, cat. #SRE0068). The membrane was UV cross-linked at 254 nm for 5 minutes on the both sides. To verify equal RNA loading, the membrane was stained with 0.02% methylene blue in 0.3 mol/L sodium acetate and scanned. After blocking with 5% BSA, the membrane was incubated with an anti-5-methylcytosine (5-mC) antibody, followed by an HRP-conjugated anti-rabbit secondary antibody. Signal detection was performed using an imaging system.

Immunohistochemistry

Immunohistochemical (IHC) staining was performed based on established protocols [13]. Briefly, tissue sections were incubated with specific primary antibodies, followed by HRP-conjugated secondary antibodies. The sections were counterstained with hematoxylin and imaged under an inverted microscope. All antibodies used are listed in Supplementary Table S4.

The IHC score was calculated by multiplying the staining intensity score by the proportion of positive cells.

Staining intensity was graded as follows: 0 (negative), 1 (weak), 2 (moderate), and 3 (strong). The percentage of positive cells was scored as: 0 (0%), 1 (<10%), 2 (10%–50%), 3 (50%–80%), and 4 (>80%). All scoring was performed independently by two experienced pathologists.

Cell proliferation assays

CCK-8 assay

Cells were seeded at a density of 2×10^3 per well in 96-well plates. At designed time points, CCK-8 reagent (MCE, cat. #HY-K0301) was added to each well. After 1 hour of incubation, the absorbance at 450 nm were detected.

EdU assay

A total of 2×10^4 cell per well were planted in 96-well plates and incubated with 50 μM EdU (UElandy, cat. #C6046M) for 2 hours. Subsequently, cells were fixed with 4% paraformaldehyde, permeabilized with 0.1% Triton X-100 for 10 minutes, and stained with EdU YF® 488 Azide for 30 minutes. Nuclei were stained with Hoechst 33342 for 30 minutes.

Colony formation assay

Cells were plated in 6-well plates at 2×10^3 cells per well and cultured for 7 days. Following incubation, cells were fixed with 4% paraformaldehyde and stained with 0.1% crystal violet.

Transwell and wound healing assays

Transwell assay

A total of 3×10^4 cells suspended in serum-free medium were seeded into the upper chamber of an 8μm Transwell insert (Corning, Washington DC, USA) and cultured for 24 hours. Cells that invaded through the membrane to the lower surface were fixed with 4% paraformaldehyde, stained with 0.1% crystal violet and counted under microscope.

Wound healing assay

Cells were grown in 6-well plates until they reached 90% confluence. A uniform wound was created in each well using a 200 μL sterile pipette tip. The same wound areas were imaged at specified time points to monitor cell migration.

RNA sequencing (RNA-Seq)

Total RNA was extracted from *NOP2*-knockdown and control T24 cells. Sequencing libraries were prepared and RNA sequencing was performed by OE Biotech (Shanghai, China) on the Illumina HiSeq X Ten platform with PE150 configuration. Differentially expressed genes (DEGs) were identified using a threshold of $|\log_2$ (fold change)

| > 1.5 and a q-value < 0.05. Functional annotation of DEGs was carried out through KEGG pathway enrichment analysis.

RNA bisulfite sequencing (RNA-Bis-Seq)

RNA-Bis-Seq of m^5C modification detection was performed by CloudSeq Biotech Inc. (Shanghai, China). Total RNA was extracted from *NOP2*-knockdown and control T24 cells. Following ribosomal RNA (rRNA) depletion, the RNA was subjected to bisulfite conversion and purification. Sequencing libraries were constructed and sequenced on an Illumina HiSeq 4000 system using 150 bp paired-end reads. After trimming 3′ adaptors and filtering low-quality reads, clean reads were aligned to the UCSC HG19 reference genome using meRanGh. Methylated sites were called with meRanCall, and differential methylation was identified using meRanCompare. The m^5C sites were annotated based on Ensembl genome features, and their distribution was visualized with the MetaPlot package in R.

RNA stability assay

BCa cells were transfected with the indicated vectors and then treated with 5 μg/mL actinomycin D (MCE, cat. #HY-17559) for specified duration. After treatment, total RNA was extracted and analyzed by RT-qPCR. The half-life of the target mRNA was calculated using non-linear regression analysis.

RNA immunoprecipitation (RIP)

The RIP assay was performed using the Magna RIP™ RNA-Binding Protein Immunoprecipitation Kit (Millipore, Burlington, USA) according to the manufacturer's instructions. Briefly, approximately 1×10^7 cells were collected and lysed in RIP lysis buffer. Magnetic beads conjugated with 5 μg of antibody against the target protein or normal IgG were incubated with the cell lysates overnight at 4 °C. The immunoprecipitated RNA–protein complexes were then treated with proteinase K buffer to digest proteins. RNA was extracted using phenol-chloroform purification and analyzed by RT–qPCR. Results were normalized to the input sample.

RNA pulldown

The RNA pulldown assay was performed with the Pierce Magnetic RNA-Protein Pull-Down Kit (Thermo Fisher Scientific, cat #20164). 3'-labeled biotin-labeled RNA probes, including 50-bp *SCD* RNA sequences with m^5C modification (SCD [m^5C]) or without m^5C modification (SCD [C]) at the same site, were synthesized (as detailed in Supplementary Table S4) and then incubated with total protein extracts from BCa cells. In brief, 50 pmol biotinylated RNA probes were incubated with 50 μL of streptavidin beads for 30 min. Then, the mix was incubated with 2 mg of protein lysates at 4°C for 60min. Following the pulldown assay, nonspecific signals were removed

by washing throughout. Protein samples were eluted by boiling with SDS buffer and subjected to western blot using anti-YBX1 antibodies.

Luciferase reporter gene assays

The wild-type (SCD^{WT}) or mutant (SCD^{MUT}) 3'-UTR sequence of SCD, containing a cytosine-to-guanine substitution at the predicted m^5C site, was cloned into a reporter vector constructed by Tran sheep Company (Shanghai, China). The resulting constructs were co-transfected with the pRL-SV40 Renilla luciferase control vector into BCa cells seeded in 24-well plates. After 48 hours, luciferase activity was measured using the Dual-Luciferase Reporter Assay Kit (Vazyme, Nanjing, China). Firefly luciferase activity was normalized to that of Renilla luciferase for each sample.

Lipid quantification assay

BCa cells with the indicated treatments were collected and washed with PBS. Lipids were extracted from cells using a Lipid Extraction Kit (Cell Biolabs, cat. #STA-612). The intracellular unsaturated fatty acids (UFAs) content was detected at OD 540 nm, and the values were calculated according to the Lipid Quantification Kit (unsaturated fatty acids; Cell Biolabs, cat. #STA-613) protocol.

Subcellular fractionation

Subcellular fractionation was carried out with the PARIS™ kit (Invitrogen, cat #AM1921) according to the manufacturer's protocol. In brief, about 5×10^6 collected cells were washed in cold PBS and lysed on ice for 10 min using 400 µl of ice-cold cell fractionation buffer. Following 500 × g centrifugation for 5 min, the supernatant (cytoplasmic fraction) was transferred to an RNase-free tube. The pellet was then lysed in an equal volume of cell disruption buffer and homogenized by vortex for 40 min on ice. After 12,000 × g centrifugation for 5 min, the supernatant (nuclear fraction) was collected. Cytoplasmic and nuclear RNAs were then extracted for RT-qPCR analysis.

Untargeted lipidomics assay

Approximately 1.2×10^7 cells were collected and washed twice with 1 ml of 0.9% normal saline. Then, 800 µl of pre-cooled methanol (−80 °C) and 320 µl ice-cold water were added, and the mixture was homogenized by shaking for 10 seconds. Metabolites were scraped and transferred to a 1.5 mL centrifuge tube. After adding 800 µL of pre-cooled chloroform, the sample was centrifuged at 14,000 × g and 4 °C for 15 minutes. A 700 µL aliquot of the lower organic phase was transferred to a new tube and dried under nitrogen gas at room temperature. The dried lipid extracts were reconstituted in 120 µL of solvent (chloroform:methanol:water, 6:3:0.5, v/v/v), vigorously

vortexed for 5 minutes, and centrifuged at 14,000 × g for 5 minutes at room temperature. Finally, 100 μL of the supernatant was transferred to a 2 mL mass spectrometry vial containing internal standards for LC–MS/MS analysis. Liquid chromatography tandem mass spectrometry (LC–MS/MS) and subsequent data processing were performed by OE Biotech (Shanghai, China).

Flow cytometry

Lipid peroxidation was assessed using 1 μmol/L C11-BODIPY 581/591 (Beyotime, cat. #S0043M), and cell viability was evaluated using propidium iodide (PI, Invitrogen, cat. #P1304MP), both according to the manufacturers' protocols. Data acquisition was performed on a CytoFLEX flow cytometer (Beckman Coulter), and results were analyzed with FlowJo software, with a minimum of 1×10^4 cells acquired per condition.

Transmission electron microscopy

Transmission electron microscopy was performed by the High-Resolution Electron Microscopy Facility at the Instrument Center of Biossci Biotechnology Co., Ltd (Wuhan, China). T24 and UMUC3 cells were cultured under indicated conditions, trypsinized, and fixed in 2.5% glutaraldehyde. The cells were then post-fixed in 1% osmium tetroxide containing 0.1% potassium ferricyanide, dehydrated through a graded ethanol series (50% to 100%), and embedded in epoxy resin. Ultrathin sections (60–80 nm) were prepared using a ultramicrotome. Sections were stained with 2% uranyl acetate in saturated alcoholic solution and lead citrate, and imaged under an 80 kV transmission electron microscope (HITACHI, Tokyo, Japan).

Cellular Fe^{2+} content assay

Intracellular Fe^{2+} levels were measured using the fluorescent indicator FerroOrange (DojinDo, cat. #F374) following the manufacturer's protocol. Briefly, *NOP2*-knockdown BCa cells were seeded at a density of 5×10^3 cells per well in 96-well plates. After incubation with 1 μM FerroOrange for 30 minutes at 37 °C, the cells were washed 3 times with PBS. Fluorescence was then measured using a Synergy Neo2 microplate reader (BioTek, Winooski, USA) at an excitation/emission wavelength of 543 nm.

Intracellular ROS level assay

Intracellular ROS levels were measured using a ROS Assay Kit (Beyotime, cat. #S0033S) according to the manufacturer's instructions. Briefly, 5×10^3 cells were seeded per well in a 96-well plate and incubated with DCFH-DA probe for 1 hour at 37 °C. Fluorescence intensity was measured at excitation/emission wavelengths of 490 nm and 525 nm, respectively, using a Synergy Neo2 microplate reader (BioTek, Winooski, USA).

Survival analysis

RNA-seq data and corresponding clinical information for GSE and TCGA-BLCA cohorts was downloaded and processed. Overall Survival (OS), Progression-Free Survival (PFS), Disease-Free Survival (DFS), and Disease-Specific Survival (DSS) were evaluated, with a maximum follow-up time set at five years (60 months). Patients were stratified into high- and low-expression groups for each gene based on an optimal cutoff value calculated through “survminer” package. The Kaplan-Meier method was used to generate survival curves for all four endpoints, and the log-rank test was applied to compare survival differences between the two groups.

Statistical analyses

All statistical analyses were conducted using GraphPad Prism 10.0, SPSS 20.0, and R version 4.3.3 (https://www.r-project.org/). Quantitative data were expressed as mean ± standard deviation (SD). Comparisons between groups for continuous variables were performed using one-way ANOVA or two-tailed Student’s t-test, as appropriate. Categorical variables were compared using the Mann-Whitney U test. Correlations were evaluated by Pearson’s correlation analysis. A P-value less than 0.05 was considered statistically significant, with significance levels indicated as follows: *$P < 0.05$, **$P < 0.01$, and ***$P < 0.001$.
